# The global burden of hospitalisation due to pneumonia caused by *Staphylococcus aureus* in the under-5 years children: A systematic review and meta-analysis

**DOI:** 10.1016/j.eclinm.2021.101267

**Published:** 2022-01-14

**Authors:** Durga Kulkarni, Xin Wang, Emma Sharland, Daniel Stansfield, Harry Campbell, Harish Nair

**Affiliations:** Centre for Global Health, Usher Institute, University of Edinburgh, Edinburgh EH8 9AG, UK

**Keywords:** Acute lower respiratory infection, Hospital, Paediatrics, Aetiology

## Abstract

**Background:**

Pneumonia is a leading cause of childhood morbidity and mortality. This study aimed to estimate the global hospitalisation due to *Staphylococcus aureus* pneumonia in under-5 children.

**Methods:**

We conducted a systematic review and meta-analysis of primary studies following the PRISMA-P guidelines. We searched Medline, Embase, Global Health, CINAHL, Global Index Medicus, Scopus, China National Knowledge Infrastructure, Wanfang, and CQvip. We included studies reporting data on *Staphylococcus aureus* pneumonia, confirmed by detection of the pathogen in sterile-site samples in under-5 hospitalised children, published in English or Chinese language and conducted between 1st January 1990 and 4th November 2021 and between 1st January 1990 and 30th September 2020, respectively. We excluded those testing upper respiratory tract samples and not reporting data on samples with other bacteria or absence of bacteria. We screened papers against pre-specified criteria, extracted data and assessed the bacteriological quality, and combined epidemiological and microbiological quality of studies using two self-designed checklists. Pooled proportions of hospitalisation episodes for *Staphylococcus aureus* pneumonia amongst all-cause pneumonia and the 95% confidence intervals were calculated using the random-effects model. The review protocol was registered on PROSPERO (CRD42021236606).

**Findings:**

Of 26,218 studies identified, thirty-five studies enroling 20,708 hospitalised pneumonia episodes were included. Out of the total hospitalised pneumonia cases in this population, the pooled proportion of Staphylococcal pneumonia cases was 3% (95% CI 2% to 4%; I^2^=96%). amongst 12 studies with higher microbiological quality, the pooled estimate was 6% (95% CI 2% to 10%; I^2^= 98%). Based on the recent global estimates of hospitalised pneumonia in this age group, the 3% and 6% estimates represent 738 thousand and 1.48 million hospitalisations in 2019, respectively. Based on the Grading of Recommendations Assessment, Development, and Evaluation (GRADE), the overall quality of evidence was considered to be moderate.

**Interpretation:**

Our findings are probably an underestimate because of the unknown and the likely limited sensitivity of current testing methods for Staphylococcal pneumonia diagnosis and widespread reported use of antibiotics before recruitment (in 46% of cases). *Staphylococcus aureus* is an important cause of hospitalisation for pneumonia in young children globally.

**Funding:**

Bill and Melinda Gates Foundation (OPP 1,172,551) through a prime award to John Hopkins University.

**Research in context**

**Evidence before this study**

We searched Medline, Embase, Global Health, CINAHL, Global Index Medicus, Scopus, China National Knowledge Infrastructure, Wanfang, and CQvip using search terms related to aetiology, pneumonia, children, and bacteria. We screened literature published between 1 January 1990 and 4 November 2021 (English databases) and 1 January 1990 and 30 September 2020 (Chinese databases). We did not find existing systematic reviews on this topic focussing on the under-5 population and in a global context based on our searches. Additionally, there seemed to be a lack of consensus within existing literature (primary studies) regarding the importance of *Staphylococcus aureus* as a cause of pneumonia in the global under-5 population.

**Added value of this study**

We only included studies establishing pneumonia diagnosis using sterile site samples and bacterial culture methods or polymerase chain reaction (PCR); and excluded studies testing specimens from the upper respiratory tract. The findings of this review suggest that 3% of hospitalised pneumonia cases (6% in studies of higher microbiological quality) in the under-5 children are due to *Staphylococcal aureus* and represents about 738,000 (1·48 million for the estimate of 6% in studies of higher microbiological quality) global hospital admissions in 2019 based on current estimates of hospitalised pneumonia in this age group. However, heterogeneity exists across estimates of different studies and these estimates are probably under-estimates due to the low sensitivity of methods and prior use of antibiotics. The overall quality of this evidence was found to be moderate. We found reports of 2 (3·4%) deaths amongst 63 cases across 6 studies, but firm conclusions on case- fatality ratio (CFR) cannot be made based on these limited data.

**Implications of all the available evidence**

*Staphylococcus aureus* is an important cause of hospitalised pneumonia in young children globally and merits more investment in research and development of novel diagnostics and therapeutics given its capacity to cause serious illness needing specific second-line antibiotic treatment and to develop multi-drug resistance. Improved diagnostic methods must be developed to help guide clinical care and improve quality of aetiological research and further epidemiological and laboratory research conducted to help inform novel antibiotic and vaccine development.

Alt-text: Unlabelled box

## Introduction

A target of the Sustainable Development Goals (SDGs) is to reduce, by 2030, the under-5 mortality to 25 deaths per 1000 live births.^1^ Although creditable progress is being made in this regard, much remains to be achieved in meeting this target. Liu et al. estimated that pneumonia caused about 762,000 deaths in children aged between 1 and 59 months and was the largest infectious killer accounting for 12.8% of all deaths in this group in 2015.^2^

The incidence and mortality due to childhood pneumonia caused by bacterial pathogens like *Streptococcus pneumoniae* and *Haemophilus influenzae* type b has reduced due to global introduction and scale-up of Hib and pneumococcal conjugate vaccines (PCV). Despite substantial reduction in childhood pneumonia mortality, the hospitalisation rates due to childhood pneumonia and pneumonia caused by specific organisms appear to be rising.^3^^,^^4^ It is estimated that there was a 187% rise in hospitalisations due to childhood pneumonia globally between the years 2000 and 2015.^3^ This is partly attributable to change in health seeking behaviour and improved access to healthcare and partly to the increase in under five population.^5^^,^^6^ *Staphylococcus aureus* is a gram-positive bacterium that can cause pneumonia that is not currently preventable by vaccination.^7^ Strains of *Staphylococcus aureus* have been gradually acquiring antimicrobial resistance in the antibiotic era causing widespread concerns.^8^ Additionally, rates of Staphylococcal pneumonia in children have been reportedly rising in recent years.^9^ The proportional contribution of pathogens (like *Staphylococcus aureus* against which vaccines currently do not exist) to pneumonia hospitalisations and deaths is likely to increase in future. Hospital admission for childhood pneumonia in Low- and Middle-Income Countries (LMICs) incurs substantial financial and productivity costs to the child and family, healthcare system, and wider society.^10^

We aimed to estimate the proportion of Staphylococcal pneumonia cases amongst all-cause pneumonia hospitalisations globally. We also intended to explore availability of data and report estimates of in-hospital case-fatality ratio (CFR) of *Staphylococcus aureus* pneumonia in children younger than 5 years. This will help improve our understanding of its importance as a cause of child pneumonia globally and inform clinical guidelines.

## Methods

### Search strategy and selection criteria

This systematic review was guided by the Preferred Reporting Items for Systematic Review and Meta-Analysis Protocols (PRISMA-P 2020) guidelines.^11^ A review protocol was registered on PROSPERO (CRD42021236606). We did not seek formal ethical approval for this systematic review as the data included in the meta-analysis were obtained from published literature.

We developed and ran comprehensive searches in six English language health-related databases (Medline, Embase, Global Health, CINAHL, Global Index Medicus, and Scopus) and three Chinese databases (China National Knowledge Infrastructure, Wanfang, and CQvip). The detailed search strategies for each database are attached in supplementary material (Appendices 1 and 2).

The searches for English language databases were reviewed by a specialist medical librarian. We also searched the reference lists of identified articles to detect any additional relevant publications. The searches were conducted on 4th and 5th November 2020 for the English and Chinese literature databases, respectively. We updated the searches in English databases on 5th November 2021. We limited our searches to studies that were conducted between 1st January 1990 and 4th November 2021 to minimise heterogeneity in studies due to developments in testing methods after 1990. We restricted our searches in the non- Chinese databases to studies published in English language. No restrictions based on geographical location were applied. The proportion of Staphylococcal pneumonia amongst all-cause pneumonia hospitalisations was our primary outcome and the in-hospital CFR of Staphylococcal pneumonia was our secondary outcome. Data for the secondary outcome were extracted, if available. However, studies were included in the review irrespective of secondary outcome data availability.

The studies had to meet the following inclusion criteria:

(1) Participants:
  I aged ≤ 60 months and hospitalised for pneumonia.
  II >50% of eligible participants having a sample from a sterile site tested for bacterial pathogens.
(2) Outcomes:
  I Primary outcome: proportion of Staphylococcal pneumonia cases (determined by the growth of *Staphylococcus aureus* in mentioned sterile site samples) amongst all-cause pneumonia hospitalisations.
  II Secondary outcome: in-hospital CFR of pneumonia where *Staphylococcus aureus* is identified.
(3) Study design: Primary data emerging from:
  I Observational studies including longitudinal, cross-sectional surveys, cohort studies, case-control studies
  II Intervention studies like drug interventions, vitamin supplementation, etc. Data from both intervention and control arms were included if bacterial pathogens were tested and isolated from a sterile site before administration of the intervention(s). If bacterial testing conducted after administration of the intervention(s) only data from the control arm were included.
(4) Case definitions: Pneumonia as defined by the World Health Organisation (WHO), pneumonia diagnosis based on clinical signs and symptoms or radiological evidence by a medical professional.^12^^,^^13^ Both community-acquired pneumonia (CAP) and nosocomial pneumonia cases included.
(5) Samples: Specimens collected from sterile sites (this includes blood, induced sputum (IS), bronchoalveolar lavage (BAL), cerebrospinal fluid, bronchial aspirate, pleural fluid, and lung fluid)
(6) Testing methods: Culture and polymerase chain reaction (PCR)
(7) Setting: Hospital in-patients
(8) Timeframe: Data collected after 1 Jan 1990 until 4 November 2021.

We added an inclusion criterion that at least 50% of eligible participants should have a sample taken from a sterile-site and tested for bacterial pathogens to minimise selection bias. The selection criteria are outlined in Appendix 3 in the supplementary material. We selected publications that included the longest study period or those reporting data from more study sites when there were more than one publication from the same study.

Titles and abstracts and full text articles in English language were screened independently by DK, ES, and DS in MS Excel after deduplication of the search results in EndNote X9. Data extractions for English language articles were conducted independently by DK, ES, and DS in MS Excel. All extractions were cross-checked by XW and in case of disagreement arbitered by HN. Chinese language publications were searched, and data extracted by a single reviewer XW. We extracted data on: country, country classification (based on World Bank income regions),^14^ location characteristic- urban/ semi-urban/ rural, hospital characteristic: primary/ secondary/ tertiary, pneumonia severity, age group, testing methods, chest x-ray findings, period of data collection, case definition used, community-acquired pneumonia (CAP) or nosocomial pneumonia, nutrition status of participants, Human immunodeficiency virus (HIV) status of participants, percentage of participants with prior administration of antibiotics before sample collection as reported by study authors, number of *Staphylococcus aureus* pneumonia cases, total number of pneumonia cases, other bacteria that were tested, number of mixed infections, mixed infections with *Staphylococcus aureus.* Pneumonia severity was determined according to the WHO classification if the authors did not classify the severity of cases in their respective studies. Severity was marked as unclear if the classification according to the WHO criteria was not possible from the case-definitions or authors’ description of the disease.^12^

We were unable to identify an established and validated quality assessment checklist that would evaluate the quality of microbiological methods along with assessing sampling methods, sample dropouts, etc. We, therefore, modified the Joanna Brigg's critical appraisal checklist^15^ and the Critical Appraisal Skills Programme (CASP) checklists^16^ to assess the methodological risk of bias in each study. This checklist was designed to address selection bias, sample selection methods, sample processing methods, etc., and is attached in the supplementary material (Appendix 8). The risk of bias assessment was performed by DK (for English language literature) and XW (for Chinese literature) and cross-checked by HC. We reconciled any disagreement by discussion between DK, XW and HC.

Finally, we, conducted a Grading of Recommendations Assessment, Development, and Evaluation (GRADE) assessment to determine the quality of evidence generated by this systematic review and meta-analysis.

### Data analysis

All the analyses were undertaken in R version 3.6.3. The outcomes were calculated as the proportion of pneumonia cases due to *Staphylococcus aureus* amongst the total number of hospitalised and sampled all-cause pneumonia cases in each included study.

For all the analyses, we utilised the random effects model to take both within- and between-study variances into account.^13^ We applied the Freeman-Tukey double arcsine transformation, and the pooled proportion was derived as back-transformed values as the weighted mean of the transformed proportions and expressed with a 95% confidence interval.^13^ We calculated the I^2^ statistic for all the statistical analyses to determine the degree of heterogeneity in included studies.

If a study employed >1 testing methods (or >1 type of sample) for a case, detection of *Staphylococcus aureus* by at least one testing method (or in at least one type of sample) was counted as a case of *Staphylococcus aureus* pneumonia and a forest plot was developed. Studies were taken to be “influential” if their exclusion resulted in significant changes in the meta-analysis results. Influential studies were identified by the influence analysis diagnostics of the proportion of Staphylococcal pneumonia cases as proposed by Viechtbauer and Cheung.^17^ We conducted sensitivity analysis by excluding such influential studies and reported the findings of this analysis.

A subgroup analysis was undertaken to study the influence of testing methods on the pooled estimates. This analysis classified studies based on the quality of culture methods and quality of methods employed for identification of organisms. The detailed classification and grading of bacteriological methods of each study and the forest plot for this analysis is provided in the supplementary material (Appendix 7).

We conducted three more sensitivity analyses. Firstly, we eliminated one group of studies testing a particular type of sample (for example, blood; IS; etc.) at a time to explore the effect of type of sample on the estimate. To this analysis, data were classified into four groups depending on the type of samples. Three studies collected and tested >1 type of sample from a patient^18^, ^19^, ^20^ and this analysis includes data for all samples that were collected and tested. Therefore, these values do not necessarily represent unique hospital episodes. Secondly, we explored the effect of level of care delivered by the healthcare centre on Staphylococcal pneumonia estimates. We graphically presented this analysis as a forest plot with studies conducted at tertiary-level centres comprising one group and those delivering primary care or with no information regarding the characteristics of healthcare setting forming another group. Lastly, we reported the findings of studies with a high epidemiological quality assessment score and those with a low epidemiological assessment score separately.

A limited number of studies reported stratified data for finer age groups within the study population. Therefore, subgroup analysis for age groups could not be conducted.

The findings for *Staphylococcus aureus* pneumonia in-hospital CFR in under-5 hospitalised children were summarised narratively because of limited data and one study with an extreme CFR estimate.

### Role of the funding source

The funder of the study had no role in study design, data collection, data analysis, data interpretation, or writing of the report.

## Results

**Figure 1:**
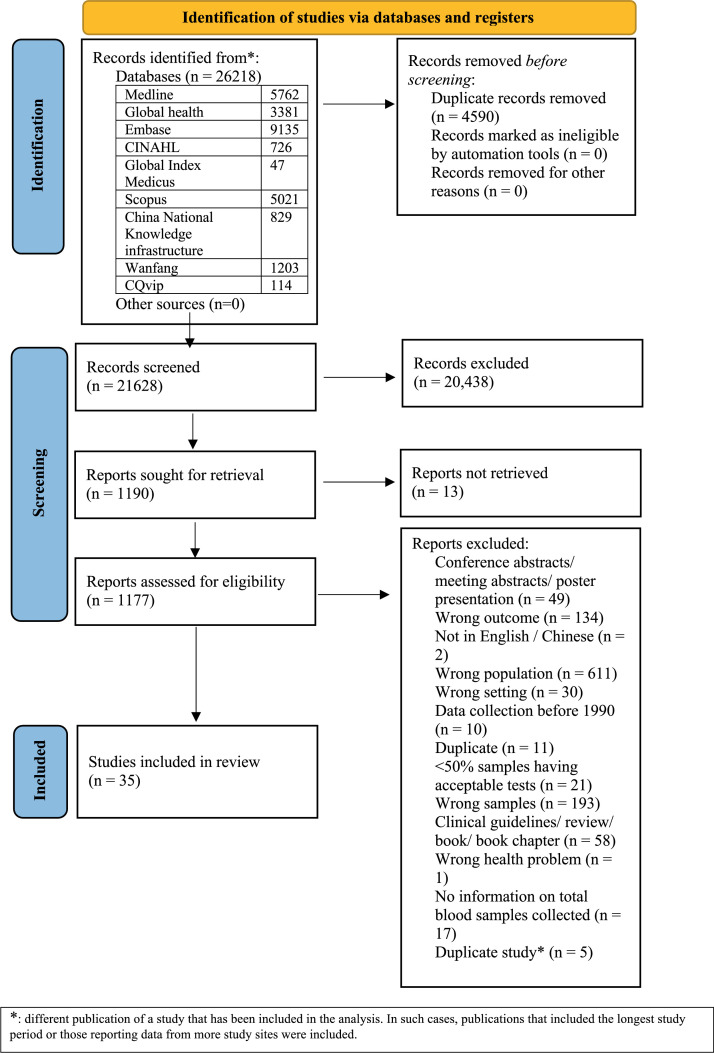
PRISMA flowchart.

We identified a total of 26,218 records of which 21,628 records were eligible for screening after de-duplication. Figure 1 reflects the flow of studies at each stage. Thirty-four studies identified via English-language database searches and one study identified via Chinese database searching were included in this systematic review and meta-analysis.^18^, ^19^, ^20^, ^21^, ^22^, ^23^, ^24^, ^25^, ^26^, ^27^, ^28^, ^29^, ^30^, ^31^, ^32^, ^33^, ^34^, ^35^, ^36^, ^37^, ^38^, ^39^, ^40^, ^41^, ^42^, ^43^, ^44^, ^45^, ^46^, ^47^, ^48^, ^49^, ^50^, ^51^, ^52^ A list of the included studies is attached in the supplementary material (Appendix 4).

The period of data collection of individual studies, their data collection locations, and testing methods are summarised in Table 1.

**Table 1:** Study characteristics.

First author (year)	Period of data collection	Duration of data collection (months)	Location name	Type of location	Type of care delivered by hospital	Country name (number of sites within the country)	Country classification	Case definition of pneumonia	Samples	Testing method
Abdelkhalig (2015)^21^	Nov 2011 to Feb 2012	4	Khartoum	unclear	tertiary	Sudan (1)	low income	clinical signs	blood	coagulase test
Adegbola (1994)^24^	Nov 1990 to Oct 1992	24	Banjul	unclear	primary	The Gambia (1)	low income	Clinical and radiological evidence of pneumonia	blood, lung, and pleural fluid	standard culture
Asghar (2008)^23^	Aug 2000 to Apr 2004	45	Dhaka, Guayaquil, Chandigarh, Mexico City, Multan and Rawalpindi, Sana'a, Lusaka	urban	tertiary	Bangladesh (1), Ecuador (1), India (1), Mexico (1), Pakistan (2), Yemen (1), Zambia (1)	mixed	WHO definition of pneumonia	blood, cerebrospinal fluid	standard culture
Bahl (1995)^24^	Sep 1991 to Jul 1992	11	New Delhi	unclear	unclear	India (1)	lower middle income	WHO definition of pneumonia	blood	standard culture
Baqui (2007)^25^	Jul 1999 to Jun 2001	24	Matlab	rural	tertiary	Bangladesh (1)	lower middle income	WHO definition of pneumonia	blood	standard culture
Bari (2014)^26^	Jan 2010 to Dec 2012	36	Lahore	unclear	unclear	Pakistan (1)	lower middle income	WHO definition of pneumonia	blood	standard culture
Barrett (2016)^27^	2002 to 2011	≈120	Beer-Sheva	unclear	unclear	Israel (1)	high income	WHO radiographic criteria of pneumonia (primary endpoint pneumonia)	blood	standard culture
Bautista-Marquez (2013)^28^	Mar 2010 to Jun 2011	16	8 sentinel hospitals in 4 states	unclear	unclear	Mexico (8)	upper middle income	WHO definition of pneumonia	blood, CSF, bronchial aspirate, synovial fluid, pleural fluid	standard culture
Benet (2017)^29^	May 2010 to March 2014	47	Phnom Penh, Beijing, Port au Prince, Pune, Lucknow, Antananarivo, Bamako, Ulaanbaatar, San Lorenzo	mixed	unclear	Cambodia (1), China (1), Haiti (1), India (2), Madagascar (1), Mali (1), Mongolia (1), and Paraguay (1)	mixed	WHO case definition of pneumonia and radiological evidence of pneumonia	blood	standard culture, PCR
Benet (2017)^30^	May 2010 to June 2014	50	Phnom Penh, Beijing, Port au Prince, Pune, Lucknow, Antananarivo, Bamako, Ulaanbaatar, San Lorenzo	mixed	unclear	Cambodia (1), China (1), Haiti (1), India (2), Madagascar (1), Mali (1), Mongolia (1), and Paraguay (1)	mixed	WHO case definition of pneumonia and radiological evidence of pneumonia	blood	standard culture, PCR
Camacho-Moreno, G. (2021)^51^	January 2016 to December 2016	12	Bogota	unclear	tertiary	Columbia	upper middle income	clinical signs and chest X-ray showing a radiological pattern compatible with bacterial pneumonia	blood	standard culture
Capeding (1994)^31^	Apr 1990 to Dec 1992	33	Muntinlupa	unclear	unclear	Phillippines (1)	lower middle income	clinical signs	blood	standard culture
Champatiray (2017)^32^	Sep 2013 to Aug 2014	12	Cuttack	unclear	unclear	India (1)	lower middle income	WHO definition of pneumonia	blood	standard culture
Ekalaksananan (2001)^33^	Aug 1992 to Nov 1994	28	Khon Kaen	unclear	unclear	Thailand (1)	upper middle income	WHO definition of pneumonia	blood	standard culture
El Mdaghri (2012)^34^	Sep 2007 to Aug 2008	12	Casablanca	unclear	unclear	Morocco (1)	lower middle income	WHO radiographic criteria of pneumonia (primary endpoint pneumonia)	blood, lung, and pleural fluid	standardised automated culture methods
Hammitt (2012)^35^	Jan 2010 to Dec 2010	12	Kilifi	rural	unclear	Kenya (1)	lower middle income	WHO definition of pneumonia	blood	standardised automated culture methods
Hasan (2006)^36^	Oct 1993 to Sep 1994	12	10 villages of Mirzapur	rural	unclear	Bangladesh (10)	lower middle income	WHO definition of pneumonia	blood	standard culture
Hijazi (1997)^37^	Aug 1993 to Jun 1994	11	Jabriya	unclear	unclear	Kuwait (1)	high income	clinical signs	blood	standard culture
Howie (2014)^18^	2007 to 2009	≈36	Fajara, Banjul, Fajikunda, Serekunda, and Brikama	unclear	unclear	The Gambia (5)	low income	WHO case definition of pneumonia and radiological evidence of pneumonia	blood; lung/ pleural aspirate	PCR; standard culture^1^
Jakhar (2017)^38^	Oct 2012 to Sep 2013	12	Delhi	urban	unclear	India (1)	lower middle income	WHO definition of pneumonia	blood	standard culture
Kurade (2018)^39^	November 2013 to May 2015	19	Western India	unclear	tertiary	India (1)	lower middle income	WHO definition of pneumonia	induced sputum	standard culture
Madhi (2000)^40^	Mar 1997 to Feb 1998	12	Soweto	urban	tertiary	South Africa (1)	upper middle income	WHO definition of pneumonia	blood	standardised automated culture methods
Moreno (2006)^41^	Jul 2002 to Dec 2003	18	Co´rdoba	unclear	tertiary	Argentina (1)	upper middle income	with clinical and radiological pneumonia and with a positive culture for a bacterial pathogen or a positive latex agglutination test in pleural fluid	blood and pleural fluid	standardised automated culture methods
Nantanda (2008)^19^	Dec 2005 to Mar 2006	4	Kampala	urban and peri‑urban	tertiary	Uganda (1)	low income	WHO definition of pneumonia	blood; induced sputum	standard culture
Nathan (2020)^20^	Oct 2014 to Oct 2016	25	Kuala Lumpur	urban	tertiary	Malaysia (1)	upper middle income	WHO definition of pneumonia	blood; induced sputum	PCR
Ngocho (2020)^42^	Jan 2017 to Dec 2017	12	Moshi municipality	unclear	unclear	Tanzania (1)	lower middle income	WHO definition of pneumonia	blood	standardised automated culture methods
Onipede (2009)^43^	Oct 2005 to Dec 2006	15	Ile-Ife	unclear	tertiary	Nigeria (1)	lower middle income	clinical signs	blood	coagulase test
PERCH (2019)^50^	Aug 2011 to Jan 2014	41	Basse, Bamako, Lusaka, Soweto, Kilifi, Dhaka and Matlab, Nakhon Phanom and Sa Kaeo	mixed	unclear	The Gambia (1), Mali (1), Zambia (1), South Africa (1), Kenya (1), Bangladesh (2), Thailand (2)	mixed	WHO definition of pneumonia	blood	standard culture
Schwarz (2010)^44^	Sep 2007 to Jul 2009	23	Ashanti Region	unclear	unclear	Ghana (1)	lower middle income	WHO definition of pneumonia	blood	standardised automated culture methods
Sigau´que (2009)^45^	Mar 2004 to Mar 2006	35	Manhica	rural	tertiary	Mozambique (1)	low income	WHO definition of pneumonia	blood	standardised automated culture methods
Thea (2017)^46^	Aug 2011 to Jan 2014	30	Basse, Bamako, Lusaka, Soweto, Kilifi, Dhaka and Matlab, Nakhon Phanom and Sa Kaeo	mixed	unclear	The Gambia (1), Mali (1), Zambia (1), South Africa (1), Kenya (1), Bangladesh (2), Thailand (2)	mixed	WHO definition of pneumonia	induced sputum	PCR
Vasconcellos (2020)^47^	2003- 2005	≈36	Salvador	unclear	unclear	Brazil (1)	upper middle income	Clinical and radiological evidence of pneumonia	blood	standardised automated culture methods
Wang (2008)^48^	Oct 2004 to May 2005	8	Beijing	unclear	unclear	China (1)	upper middle income	Clinical and radiological evidence of pneumonia	blood	standard culture
Yadav (2021)^52^	March 2016 to August 2017	18	Etawah	rural	tertiary	India (1)	lower middle income	WHO definition of pneumonia	blood	standard culture
Zhao (2018)^49^	Oct 2016-Feb 2018	17	Guangdong	urban	tertiary	China (1)	upper middle	Clinical and radiological evidence of pneumonia	bronchoalveolar lavage fluid	standard culture and PCR

1 blood subjected to PCR and lung/ pleural aspirate subjected to standard culture.

The distribution of studies according to pneumonia severity is illustrated in the supplementary material in Appendix 5a.

The classification of pneumonia according to acquisition of infection in the community or healthcare setting was clear in twenty-one studies and unclear in fourteen studies (Appendix 5b). None of the studies explicitly focussed on nosocomial infections. Amongst the included studies, five studies^23^^,^^47^, ^48^, ^49^, ^50^ excluded HIV positive participants and ten studies^18^^,^^19^^,^^29^^,^^30^^,^^35^^,^^40^^,^^42^^,^^44^, ^45^, ^46^ included HIV positive children. The HIV status of participants was unknown in twenty studies.^20^, ^21^, ^22^^,^^24^, ^25^, ^26^, ^27^, ^28^^,^^31^, ^32^, ^33^, ^34^^,^^36^, ^37^, ^38^, ^39^^,^^41^^,^^43^^,^^51^^,^^52^

Hijazi et al. excluded malnourished children.^37^ Malnourished children were included in eighteen studies.^19^, ^20^, ^21^, ^22^, ^23^, ^24^, ^25^^,^^29^, ^30^, ^31^, ^32^^,^^35^^,^^38^^,^^40^^,^^42^^,^^45^^,^^46^^,^^50^ The nutrition status of children was unclear in the remaining sixteen studies.^18^^,^^26^, ^27^, ^28^^,^^31^^,^^33^^,^^36^^,^^39^^,^^41^^,^^43^^,^^44^^,^^47^^,^^49^^,^^51^^,^^52^

Antibiotic administration before admission was widely prevalent, although all studies seemed to ensure that antibiotics were not administered in the hospital until samples for bacterial testing were collected. Data on this aspect were generally collected by conducting interviews with parents or testing urine for antibiotics. Within the available data, the cumulative average prior antibiotic usage was found in about 46% of participants. The study by Hasan et al. was the only study that reportedly did not have a single participant with the administration of antibiotics before sample collection (inside or outside hospital setting).^36^ The highest proportion was found to be about 84% in the study by Hammit et al.^35^

A total of thirteen infections with *Staphylococcus aureus* from three studies were reportedly mixed infections.^18^^,^^20^^,^^37^ Viruses were the most common co-infectants followed by *Streptococcus pneumoniae* and *Haemophilus influenzae type b.* Six studies reported the absence of evidence of any mixed infections with *Staphylococcus aureus.*^27^^,^^28^^,^^31^^,^^32^^,^^48^^,^^50^ Information about mixed infections was lacking in the remaining studies.

This meta-analysis was based on 20,708 hospitalisations from thirty-four studies.^18^, ^19^, ^20^, ^21^, ^22^, ^23^, ^24^, ^25^, ^26^, ^27^, ^28^^,^^30^, ^31^, ^32^, ^33^, ^34^, ^35^, ^36^, ^37^, ^38^, ^39^, ^40^, ^41^, ^42^, ^43^, ^44^, ^45^, ^46^, ^47^, ^48^, ^49^, ^50^, ^51^, ^52^ Of these, 20,707 samples were collected from unique hospitalisation episodes as one study derived and tested two samples from a single patient for a particular testing method at different time points within the same hospital admission.^18^ Two publications were based on the same study.^29^^,^^30^ We, therefore, excluded the study that reported the findings for a subsample of the main study for the hospitalisation analysis.^29^ This study was, however, retained in this review (as depicted in Figure 1) for CFR analysis as data for CFR analysis were not reported in the main study.^30^

Three studies collected a sample from >1 sterile-site for individual patients.^18^, ^19^, ^20^ Two studies employed >1 testing methods for the detection of pneumonia-causing pathogen(s) in individual patients.^30^^,^^49^ The remaining studies collected samples from unique hospital admissions.

**Figure 2:**
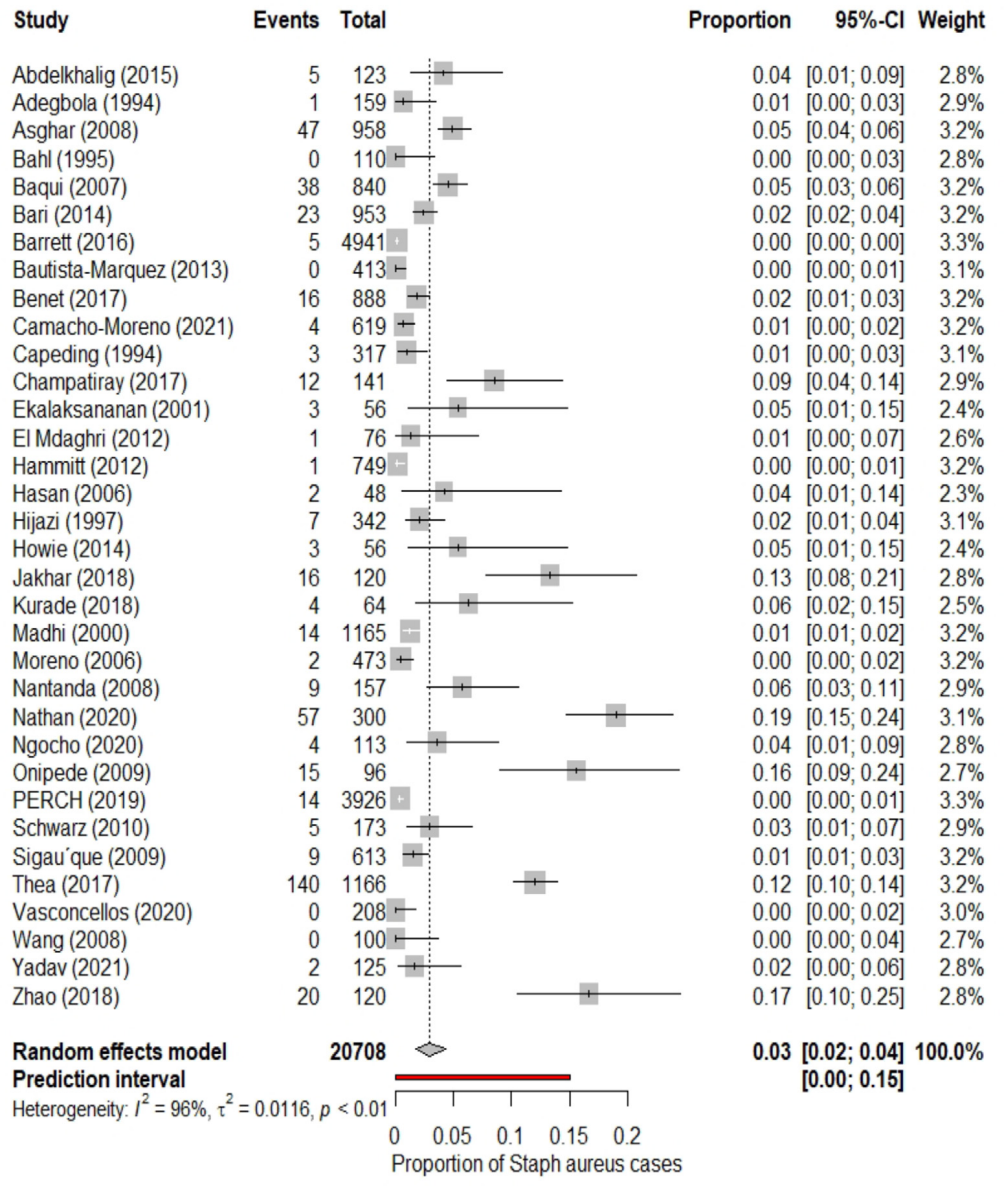
Forest plot for the pooled proportion of Staphylococcus aureus pneumonia in under-5 children hospitalised for pneumonia.

We found that out of the total hospitalised pneumonia cases in this population, the pooled proportion of Staphylococcal pneumonia cases was 3% (95% CI 2% to 4%; I^2^=96%). The 95% prediction intervals were 0% to 15%. The estimates in the included studies ranged from 0% to 19%. These findings are illustrated in Figure 2. Thus, 3% of the total pneumonia cases in the under-5 years age group that required hospitalisation were attributable to *Staphylococcus aureus*. The I^2^ value (96%) suggested that there was a high degree of heterogeneity across the findings of different studies. We plotted the proportion of *Staphylococcus aureus* cases versus the sample size of individual studies. But the evidence of a clear relationship between the two variables was found to be lacking (supplementary material).

We did not identify any influential studies such that their exclusion from the analysis would lead to significant changes in the pooled estimates (Appendix 6). Similar findings are reflected from the forest plot (Figure 2) that shows at least some degree of overlap of the 95% confidence intervals between studies and the absence of extreme effect sizes. However, five studies had a covariance ratio of <1.^20^^,^^38^^,^^43^^,^^46^^,^^49^ This meant that the resultant heterogeneity or imprecision of estimates was mainly contributed by these studies. The exclusion of these five studies from the analyses gave a pooled estimate of 2% (95% CI 1% to 2%; I^2^=92%) that was based on 18,906 samples.

**Figure 3:**
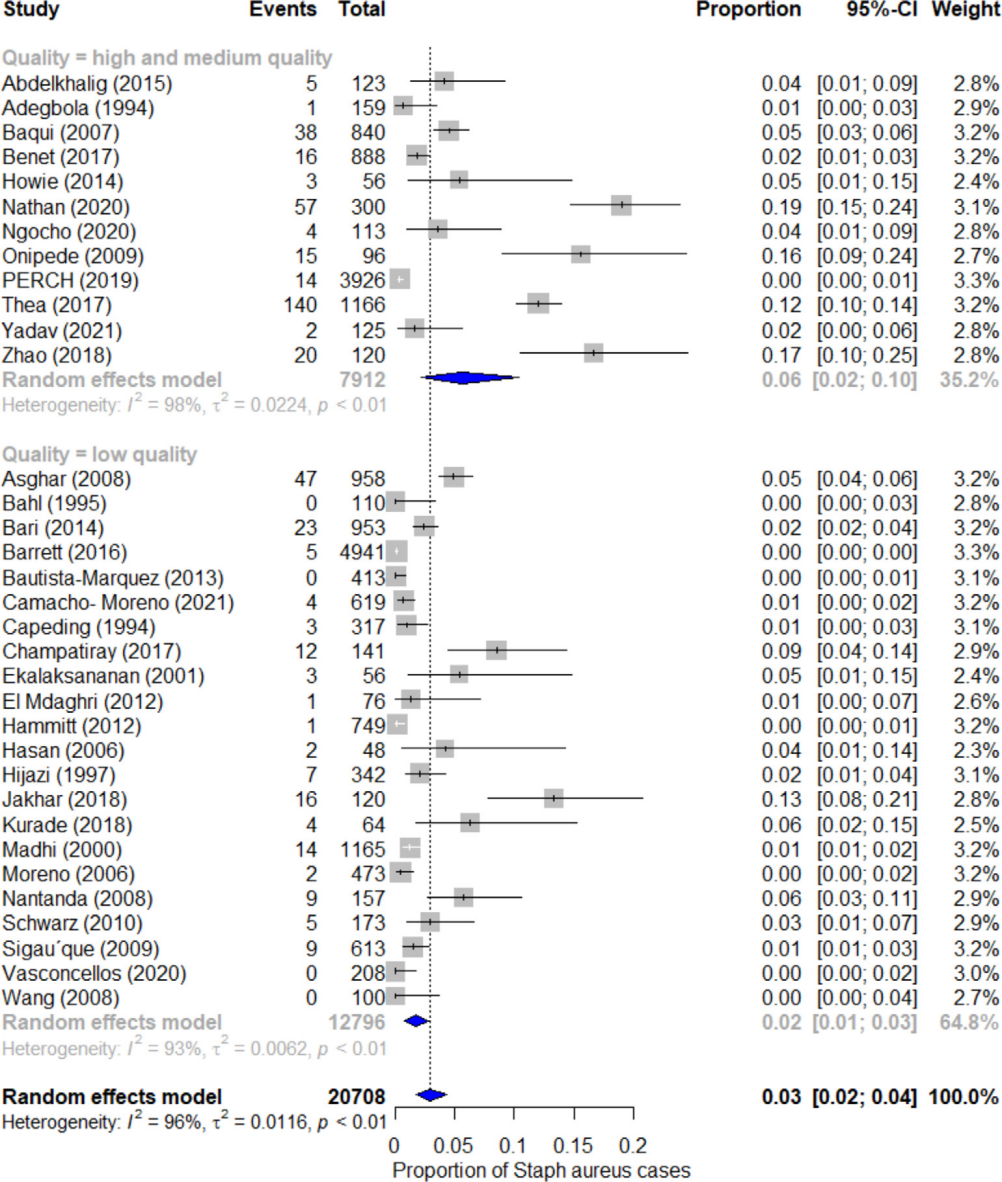
Forest plot for the pooled proportion of Staphylococcus aureus pneumonia in under-5 children hospitalised for pneumonia by subgroup: Bacteriological methods quality.

Figure 3 shows the results of the sub-group analysis based on the quality of bacteriological testing methods. The high and medium quality studies (*n* = 12) accounted for 7912 episodes of hospitalisations and gave a pooled estimate of the proportion of staphylococcal pneumonia cases as 6% (95% CI 2% to 10%; I^2^= 98%).^18^^,^^20^, ^21^, ^22^^,^^25^^,^^30^^,^^42^^,^^43^^,^^46^^,^^49^^,^^50^^,^^54^ Low quality studies (*n* = 22) accounted for 12,796 hospitalisation episodes and gave a pooled estimate of 2% (95% CI 1% to 3%, I^2^= 93%).^19^^,^^23^^,^^24^^,^^26^, ^27^, ^28^^,^^31^, ^32^, ^33^, ^34^, ^35^, ^36^, ^37^, ^38^, ^39^, ^40^, ^41^^,^^44^^,^^45^^,^^47^^,^^48^^,^^51^

The pooled estimate of the proportion of Staphylococcal pneumonia cases amongst hospitalised pneumonia cases in under-5 children after eliminating each subgroup of the type of samples is summarised in Table 2. A pooled estimate of 0·04 was obtained when blood samples were eliminated from the analysis. The pooled estimate was 0·03 after the removal of each one of the other samples. The I^2^ values showed that each of these four estimates continued to be associated with substantial heterogeneity. Blood was the most widely collected and tested sample across studies and BAL the least.

**Table 2:** Summary of findings of the sensitivity analysis by type of sample.

Subgroup eliminated from analysis	Number of participants	Pooled estimate	95% CI	I^2^
None	21,946	3%	(2%, 4%)	96%
Blood	3959	4%	(1%, 8%)	95%
Induced sputum	20,259	3%	(2%, 5%)	95%
Bronchoalveolar lavage	21,706	3%	(2%, 4%)	96%
Mixed (blood, cerebrospinal fluid, bronchial aspirate, pleural fluid, and lung fluid	19,914	3%	(2%, 5%)	96%

We observed in our sensitivity analysis that studies (*n* = 13) conducted at centres delivering tertiary-level care gave a pooled estimate of 5% (95% CI 2% to 8%; I^2^ = 95%) and those (*n* = 21) delivering unknown or other level care gave a pooled estimate of 2% (95% CI 1% to 3%; I^2^= 96%) (Appendix 9).

The summary of the combined epidemiological and microbiological quality assessment of the included studies is attached in the supplementary material (Appendix 8). Our sensitivity analysis showed that studies with a score ≥5 out of 12 (*n* = 15) accounted for 9939 pneumonia hospitalisations and resulted in a pooled estimate of 5% (95% CI 3% to 9%; I^2^= 97%); while those with a score of < 5 out of 12 (*n* = 19) represented total 10,769 pneumonia hospitalisations and gave a pooled estimate of 1% (95% CI 1% to 3%; I^2^= 92%).

McAllister et al. (2019) estimated there were 16·4 million episodes of hospitalised pneumonia globally in under-5 children in 2015.^3^ Applying the 3% (our estimate based on all included studies) and 6% (our estimate based on data restricted to studies of high quality only) to the16·4 million all- pneumonia hospitalisations in 2015 translates to 492 thousand and 984 thousand Staphylococcus pneumonia hospitalisations respectively in 2015. Their analysis also showed a 187% rise in child pneumonia hospitalisations over 15 years from 2000 to 2015.^3^ We extrapolated this 12.5% increment each year or a cumulative 50% rise from 2015 to 2019 (assuming a similar year-on-year rise in previous years) and calculated that the total number of global hospitalisations due to Staphylococcus pneumonia would be about 738 thousand (according to our estimate of 3% based on all included studies) or about 1·48 million (according to our estimate of 6% based on data restricted to studies of high quality only) in 2019.

We identified six studies with data on in-hospital CFR of Staphylococcal pneumonia in children younger than 5 years hospitalised for pneumonia.^26^^,^^29^^,^^37^^,^^38^^,^^44^^,^^51^ There were 2 (3.4%) reported deaths amongst 63 Staphylococcal pneumonia cases across the six studies. These studies comprised of twenty-three, eight, seven, sixteen, five, and four Staphylococcal cases.^26^^,^^29^^,^^37^^,^^38^^,^^44^^,^^51^ The study by Bari et al. reported two deaths in twenty-three Staphylococcal pneumonia cases in the under-5 hospitalised children in their study resulting in a CFR of 0.09.^26^

Based on principles from the Grading of Recommendations Assessment, Development, and Evaluation (GRADE), the quality of evidence generated by this systematic review and meta-analysis is regarded as moderate (considering the risk of bias, imprecision, inconsistency, indirectness, and publication bias involved in included studies).

## Discussion

Our results suggest that 3% of the pneumonia cases in children <5 years hospitalised for all-cause pneumonia are caused by *Staphylococcus aureus* with a higher estimate of 6% being found in studies with a higher quality of microbiological methods. This represents 738 thousand (3%) or 1.48 million (6%) hospital admissions in 2019 based on current estimates of hospitalised pneumonia in this age group.^3^ This is likely to the represent lower bound of the true proportion as the testing methods based on sterile-site samples have limited sensitivity for the detection of Staphylococcal pneumonia and the high reported use of antibiotics before recruitment. There was, also, substantial heterogeneity between studies. Good quality studies with larger sample sizes (across many hospitals) reporting CFR data and employing microbiological testing methods with high specificity like coagulase testing or PCR are needed.

Some extent of the heterogeneity in the estimates across studies is likely to result from the differences in case definitions and radiographic features in different studies. However, all studies identified children with pneumonia or acute lower respiratory infection (ALRI) that was considered severe enough for hospital admission by the local clinical team. None of the included studies reported data on local nosocomial outbreaks caused by *Staphylococcus aureus*. Further, except the study by Adegbola et al., for which we extracted data only for malnourished children because the sampling of well-nourished children was unclear, no other studies recruited specific subgroups of patients.^22^ We, thus, believe that our estimates provide a general representation of hospitalised pneumonia at the study sites.

Any variability in findings across studies is unlikely to be due to seasonal variations as thirty studies collected data for at least 12 consecutive months (average data collection period=26 months).^18^^,^^20^^,^^22^^,^^23^^,^^25^, ^26^, ^27^, ^28^, ^29^, ^30^, ^31^, ^32^, ^33^, ^34^, ^35^, ^36^^,^^38^, ^39^, ^40^, ^41^, ^42^, ^43^, ^44^, ^45^, ^46^, ^47^^,^^49^^,^^50^^,^^51^^,^^52^ However, none of the included studies explicitly reported data on the seasonality of *Staphylococcus aureus* pneumonia.

Early pneumonia diagnosis with the detection of the causal pathogen/s is critical to initiate effective therapy, ensure recovery and minimise the burden of childhood pneumonia and combat antimicrobial resistance.^53^ However, accurate detection of pneumonia-causing pathogens is very challenging. Sterile-site specimens are considered the “gold standard” for the detection of bacterial pathogens causing pneumonia but are recognised to have poor sensitivity.^54^^,^^55^ Upper respiratory samples are convenient to obtain but have a low specificity as this site harbours multiple pathogens as colonisers, regardless of any clinical symptoms.^56^ This is the main reason why we excluded studies based on these data. The convenience of obtaining a blood sample compared to others seems to make blood the most collected and tested sample. Adegbola et al. and Kurade et al. discussed the challenges and incidence of complications arising during the collection of IS and lung aspirate as samples in their study.^22^^,^^39^ Contamination of IS samples with upper respiratory specimens is another commonly encountered challenge^56^ The relatively higher estimate measures by Thea et al. and Nathan et al. with IS samples are likely to be a result of such contamination.^46^^,^^20^ Nathan et al. conducted blood as well as IS PCR for a sample of 300 children.^20^ *Staphylococcus aureus* grew in four blood samples and fifty-six IS samples. This further emphasises that the findings of IS samples need to be interpreted with extreme caution.

*Staphylococcus aureus* is known to be a common cause of complicated pneumonia in the paediatric population and that can lead to specific radiological patterns like cavitations, abscesses and parapneumonic effusions. Although we recognise that radiological evidence is not sufficient to determine the aetiology of pneumonia, it is useful to note that such radiological features can help to suggest the aetiology. This is particularly important considering the poor yield of microbiological methods and lack of resources in low resource settings. Radiological evidence may, thus, be used to prioritise or select cases for microbiological testing as done by Camacho et al.^51^

The increasing availability of post-mortem data from child mortality studies is helping us to better understand the complex interplay of pathogens and syndromes leading to death. One such example is the provision of structured descriptions of cause of death at sentinel sites in Asia and Africa as part of the CHAMPS network that identify the specific role of respiratory pathogens in causing death.^57^ We have excluded such studies or reports from our systematic review because these studies would not have included a consecutive sample of all pneumonia cases reported to the health facilities involved in the study and would have looked at a very specific population (children who have died). But future research is likely to benefit by inclusion of such data.

Blood samples were the most widely tested in included studies. Previous studies suggest that the sample volume of blood in childhood pneumonia is typically low and that an increase in blood volume sample is associated with an increase in bacterial yield in children.^58^^,^^59^ Variability in these methods and level of prior antibiotic use may be key factors in the high levels of heterogeneity found (interpreted from I^2^ values). Thus, these findings should be interpreted with a caveat.

Malnutrition and HIV infection in children with pneumonia are associated with an increased risk of treatment failure and high in-hospital CFR.^60^ A systematic review revealed that Klebsiella species and *Staphylococcus aureus* were the most common pneumonia-causing pathogens in severely malnourished children.^61^ There have been a few contrasting reports from high-income and low- and middle-income settings regarding the colonisation and incidence of infections with multi-drug resistant *Staphylococcus aureus* in HIV-positive children.^62^ Reports from low- and middle-income settings indicate that multi-drug resistant *Staphylococcus aureus* strains are more common in HIV-positive children than those without HIV.^63^ It may, thus, be assumed that malnutrition and HIV status of participants in individual studies contributed towards the heterogeneity in the estimates.

Prior antibiotic usage was found to be prevalent in most studies. About 46% of the review population had a history of antibiotic use. We believe that the burden of *Staphylococcus aureus* pneumonia in the study population is underestimated because of suppression of pathogenic growth in samples due to such antibiotic exposure resulting in lower sensitivity of culture-based methods. Additionally, antibiotics were often administered by parents without medical advice. Thus, the appropriateness of drug selection and dosage remains uncertain.

The findings of our sensitivity analysis based on the level of health facilities showed that *Staphylococcus aureus* pneumonia was more common in tertiary-level health facilities. This may be due to it causing more serious infections or being more common in children referred with co-morbidities or due to the availability of resources to conduct higher quality of microbiological investigation at this level.

Microbiological methods for the culture and identification of *Staphylococcus aureus* are challenging and methodological quality varies widely. Sample contamination can suppress the growth of the true pathogen.^25^ It is reported that of total blood cultures growing staphylococci in clinical practice, around 60–80% contain coagulase-negative staphylococci (CoNS), of which Staphylococcus epidermidis is pre-dominant.^64^ Since these species are rarely considered to be clinically significant in immunocompetent individuals,^65^ techniques like PCR or coagulase testing which can differentiate between the different species of Staphylococci and between the pathogenic and coagulase-positive Staphylococcus aureus and CoNS must be employed.^66^ We, therefore, undertook a subgroup analysis to explore the impact of bacteriological methods on our estimates. Studies with high and medium quality bacteriological methods (*n* = 12) reported a higher estimate (6%) compared to the estimate (2%) reported by those with lower quality bacteriological methods (*n* = 22). Another exploratory analysis (Appendix 7) showed that the estimates were 6%, 6% and 2% in studies with high (*n* = 6), medium (*n* = 6), and low quality (*n* = 22) bacteriology. However, evidence of heterogeneity (determined by I^2^ values) was found within each subgroup for both analyses.

Our sensitivity analysis also suggested that studies with higher combined epidemiological and microbiological methodological quality gave a higher pooled estimate (5%) of Staphylococcal pneumonia compared to low quality studies (1%). These differences might have resulted due to higher quality studies employing better bacteriological methods and taking precautions to avoid sample contamination. However, the confidence interval for the estimate was wide (3% to 9%) and there was substantial heterogeneity across studies (I^2^= 97%). This reflects a high degree of uncertainty in our pooled estimate.

We identified another systematic review and meta-analysis conducted on studies reported from China which reported that *Staphylococcus aureus* was found in about 3·9% in Chinese children.^67^ This estimate is slightly higher than our main estimate and this may be because it included results derived from upper respiratory samples, that are known to have low specificity in establishing the causal agent of pneumonia. Differences likely exist in Staphylococcus epidemiology across populations which lead to variation in estimates across studies reported from different countries.

The in-hospital CFR analysis of this review was based on only 2 (3·4%) reported deaths amongst 63 cases derived from six studies with limited sample sizes.^26^^,^^29^^,^^37^^,^^38^^,^^44^^,^^51^ Additionally, these studies do not include any follow-up after discharge. Relevant data were lacking in the remaining studies. A non-zero CFR value (0.09) was only reported by one study.^26^ It is important to bear in mind that CFR data identified in this systematic review are extremely limited and so no firm conclusions on in-hospital CFR can be drawn from these data.

Our study has several advantages. The study was guided by PRISMA-P guidelines and followed a pre-determined protocol. A wide variety of English and Chinese literature databases was searched to identify relevant publications. However, our study is limited by substantial heterogeneity in case-definitions, samples, and testing methods that is likely to have affected our pooled estimates. This systematic review lacked the power to detect any association between *Staphylococcus aureus* and co-infections with other bacterial or viral pathogens because of lack of information in included studies. We have excluded some studies with good microbiological screening (such as the study by Sigauque et al.^68^) because these studies do not report the proportion of *Staphylococcus aureus* in pneumonia cases but commonly report the proportion of *Staphylococcus aureus* in bacteraemia cases and the proportion of pneumonia (and other presentations) amongst these bacteraemia cases.

Although childhood pneumonia caused due to *Staphylococcus aureus* is not yet preventable by vaccines, there are several risk factors associated with pneumonia incidence and severity that are modifiable. These risk factors are known to be associated with childhood pneumonia and can be expected to have an association with Staphylococcal pneumonia specifically. These include childcare practices contributing to malnutrition or antibiotic abuse and environmental factors like indoor air pollution.^3^ Implementation of appropriate prevention strategies by identifying the prevalence of these risk factors is necessary to reduce the hospitalisation burden in children.

In summary, *Staphylococcus aureus* is an important cause of global pneumonia hospitalisation in the under-5 children. More investment in research, diagnostics, and therapeutics is warranted given its capacity to cause serious illness and develop antibiotic resistance.

### Contributors

HC and HN conceptualised the study. DK, HC, and HN developed the study protocol. DK undertook the searches. DK, ES, and DS conducted the screening for the English language publications. XW undertook the searches and the screening for the Chinese language publications. DK, ES, and DS conducted data extraction for English language studies and XW cross-checked all the entries. XW performed the data extraction for Chinese language studies. All authors had access to data. Quality assessment was performed by DK, HC, and XW. HC, HN, and DK led the data interpretation with a substantial contribution from XW. DK wrote the first draft report with input from XW and HC. All other authors revised the report critically for important intellectual content. All authors have read and approved the final version of the report. All authors had full access to all the data in the study and had final responsibility for the decision to submit for publication.

## Declaration of interests

DK reports fees from the Bill and Melinda Gates Foundation via the University of Edinburgh. HC reports grants and personal fees from WHO, Bill & Melinda Gates Foundation, Johns Hopkins University, Sanofi via the University of Edinburgh during the conduct of the study. HN reports grants and personal fees from Bill & Melinda Gates Foundation during the conduct of the study; grants from Innovative Medicines Initiative, WHO, UK National Institute for Health Research, grants and personal fees from Foundation for Influenza Epidemiology, Sanofi, personal fees from Janssen, AbbVie, and Reviral via the University of Edinburgh outside the submitted work. All other authors report no conflicts.

## Appendix Supplementary materials

Image, application 1

Image, application 2

## References

[1] United Nations. Resolution adopted by the General Assembly on 25 September 2015. United Nations General Assembly 2015. https://www.un.org/en/development/desa/population/migration/generalassembly/docs/globalcompact/A_RES_70_1_E.pdf (accessed 07 Jan 2022).

[2] Liu L., Oza S., Hogan D., Chu Y., Perin J., Jhu Z. (2016). Global, regional, and national causes of under-5 mortality in 2000–15: an updated systematic analysis with implications for the Sustainable Development Goals. Lancet.

[3] McAllister D.A., Liu L., Shi T. (2019). Global, regional, and national estimates of pneumonia morbidity and mortality in children younger than 5 years between 2000 and 2015: a systematic analysis. Lancet Glob Health.

[4] Carrillo-Marquez M.A., Hulten K.G., Hammerman W., Lamberth L., Mason E.O., Kaplan S.L. (2011). Staphylococcus aureus pneumonia in children in the era of community-acquired methicillin-resistance at texas children’s hospital. Pediatr Infect Dis J.

[5] Geldsetzer P., Williams T.C., Kirolos A. (2014). The recognition of and care seeking behaviour for childhood illness in developing countries: a systematic review. PLoS ONE.

[6] (2019). https://population.un.org/wpp/Download/Standard/Population/.

[7] Walker C.L.F., Rudan I., Liu L. (2013). Global burden of childhood pneumonia and diarrhoea. Lancet.

[8] Álvarez A., Fernández L., Gutiérrez D., Iglesias B., Rodríguez A., García P. (2019). Methicillin-resistant Staphylococcus aureus in hospitals: latest trends and treatments based on bacteriophages. J Clin Microbiol.

[9] Carrillo-Marquez M.A., Hulten K.G., Hammerman W., Lamberth L., Mason E.O., Kaplan S.L. (2011). Staphylococcus aureus pneumonia in children in the era of community-acquired methicillin-resistance at texas children's hospital. Pediatr Infect Dis J.

[10] Mumford V., Baysari M.T., Kalinin D. (2018). Measuring the financial and productivity burden of paediatric hospitalisation on the wider family network. J Paediatr Child Health.

[11] Page M.J., McKenzie J.E., Bossuyt P.M. (2021). The PRISMA 2020 statement: an updated guideline for reporting systematic reviews. BMJ.

[12] World Health Organisation (2010).

[13] Cherian T., Mulholland E.K., Carlin J.B. (2005). Standardized interpretation of paediatric chest radiographs for the diagnosis of pneumonia in epidemiological studies. Bull World Health Organ.

[14] The World Bank. World bank country and lending groups. 2021. https://datahelpdesk.worldbank.org/knowledgebase/articles/906519-world-bank-country-and-lending-groups (accessed 26 Aug 2021).

[15] Munn Z., Moola S., Riitano D., Lisy K. (2014). The development of a critical appraisal tool for use in systematic reviews addressing questions of prevalence. International Journal of Health Policy and Management.

[16] Critical Appraisal Skills Programme. CASP checklists. 2021. https://casp-uk.net/casp-tools-checklists/ (accessed 10 Nov 2021).

[17] Viechtbauer W., Cheung M.W.L. (2010). Outlier and influence diagnostics for meta-analysis. Res Synth Methods.

[18] Howie S.R., Morris G.A., Tokarz R. (2014). Etiology of severe childhood pneumonia in the Gambia, West Africa, determined by conventional and molecular microbiological analyses of lung and pleural aspirate samples. Clin Infect Dis.

[19] Nantanda R., Hildenwall H., Peterson S., Kaddu-Mulindwa D., Kalyesubula I., Tumwine K. (2008). Bacterial aetiology and outcome in children with severe pneumonia in Uganda. Ann Trop Paediatr.

[20] Nathan A.M., Teh C.S.J., Jabar K.A. (2020). Bacterial pneumonia and its associated factors in children from a developing country: a prospective cohort study. PLoS ONE.

[21] Abdelkhalig S.M., Mahgoub E.G., Soghaier M.A. (2015). Viral and bacterial acute lower respiratory tract infections in Khartoum children emergency hospital in 2012. J Public Health Epidemiol.

[22] Adegbola R.A., Falade A.G., Sam B.E. (1994). The etiology of pneumonia in malnourished and well-nourished Gambian children. Pediatr Infect Dis J.

[23] Asghar R., Banajeh S., Egas J. (2008). Chloramphenicol versus ampicillin plus gentamicin for community acquired very severe pneumonia among children aged 2-59 months in low resource settings: multicentre randomised controlled trial (SPEAR study). BMJ.

[24] Bahl R., Mishra S., Sharma D., Singhal A., Kumari S. (1995). A bacteriological study in hospitalized children with pneumonia. Ann Trop Paediatr.

[25] Baqui A.H., Rahman M., Zaman K. (2007). A population-based study of hospital admission incidence rate and bacterial aetiology of acute lower respiratory infections in children aged less than five years in Bangladesh. J Health Popul Nutr.

[26] Bari A., Zafar A., Mushtaq A., Ahmad T.M., Ejaz I., Ejaz H. (2014). Disease pattern and bacteriological profile of childhood pneumonia. Pak Paediatr J.

[27] Barrett C., Ben-Shimol S., Greenberg D. (2016). Differences between radiologically confirmed pneumonia with and without pleural fluid in hospitalized children younger than 5 years in southern Israel. Clin Pediatr.

[28] Bautista-Márquez A., Richardson V., Ortiz-Orozco O. (2013). Prevalence of pneumococcal disease, serotype distribution, and antimicrobial susceptibility in Mexican children younger than 5 years of age. Arch Med Res.

[29] Bénet T., Picot V.S., Awasthi S. (2017). Severity of pneumonia in under 5-year-old children from developing countries: a multicenter, prospective, observational study. Am J Trop Med Hyg.

[30] Bénet T., Sánchez Picot V., Messaoudi M. (2017). Microorganisms associated with pneumonia in children< 5 years of age in developing and emerging countries: the GABRIEL pneumonia multicenter, prospective, case-control study. Clin Infect Dis.

[31] Capeding M.R.Z., Sombrero L.T., Paladin F.J., Suzuki H., Numazaki Y., Saniel M. (1994). Etiology of acute lower respiratory infection in Filipino children under five years. Southeast Asian J Trop Med Public Health.

[32] Champatiray J., Satapathy J., Kashyap B., Mondal D. (2017). Clinico-aetiological study of severe and very severe pneumonia in two months to five years children in a tertiary health care centre in Odisha, India. J Clin Diagn Res JCDR.

[33] Ekalaksananan T., Pientong C., Kongyingyoes B., Pairojkul S., Teeratakulpisarn J., Heng S. (2001). Etiology of acute lower respiratory tract infection in children at srinagarind hospital, Khon Kaen, Thailand. Southeast Asian J Trop Med Public Health.

[34] El Mdaghri N., Jilali N., Belabbes H., Jouhadi Z., Lahssoune M., Zaid S. (2012). Epidemiological profile of invasive bacterial diseases in children in Casablanca, Morocco: antimicrobial susceptibilities and serotype distribution. EMHJ East Mediterr Health J.

[35] Hammitt L.L., Kazungu S., Morpeth S.C. (2012). A preliminary study of pneumonia etiology among hospitalized children in Kenya. Clin Infect Dis.

[36] Hasan K., Jolly P., Marquis G. (2006). Viral etiology of pneumonia in a cohort of newborns till 24 months of age in Rural Mirzapur, Bangladesh. Scand J Infect Dis.

[37] Hijazi Z., Pacsa A., El-Gharbawy F. (1997). Acute lower respiratory tract infections in children in Kuwait. Ann Trop Paediatr.

[38] Jakhar S.K., Pandey M., Shah D. (2018). Etiology and risk factors determining poor outcome of severe pneumonia in under–five children. Indian J Pediatr.

[39] Kurade A., Dhanawade S., Shetti S. (2018). Induced Sputum as a Diagnostic Tool in Pneumonia in Under Five Children—A Hospital-based Study. J Trop Pediatr.

[40] Madhi S.A., Petersen K., Madhi A., Khoosal M., Klugman K.P. (2000). Increased disease burden and antibiotic resistance of bacteria causing severe community-acquired lower respiratory tract infections in human immunodeficiency virus type 1-infected children. Clin Infect Dis.

[41] Moreno L., Krishnan J.A., Duran P., Ferrero F. (2006). Development and validation of a clinical prediction rule to distinguish bacterial from viral pneumonia in children. Pediatr Pulmonol.

[42] Ngocho J.S., Horumpende P.G., de Jonge M.I., Mmbaga B.T. (2020). Inappropriate treatment of community-acquired pneumonia among children under five years of age in Tanzania. Int J Infect Dis.

[43] Onipede A.O., Onayade A.A., Elusiyan J.B. (2009). Invasive bacteria isolates from children with severe infections in a Nigerian hospital. J Infect Dev Ctries.

[44] Schwarz N.G., Sarpong N., Hünger F. (2010). Systemic bacteraemia in children presenting with clinical pneumonia and the impact of non-typhoid salmonella (NTS). BMC Infect Dis.

[45] Sigaúque B., Roca A., Bassat Q. (2009). Severe pneumonia in Mozambican young children: clinical and radiological characteristics and risk factors. J Trop Pediatr.

[46] Thea D.M., Seidenberg P., Park D.E. (2017). Limited utility of polymerase chain reaction in induced sputum specimens for determining the causes of childhood pneumonia in resource-poor settings: findings from the pneumonia etiology research for child health (PERCH) study. Clinical Infectious Diseases.

[47] Vasconcellos Â.G., Clarêncio J., Andrade D., Araújo-Neto C.A., Barral A., Nascimento-Carvalho C.M. (2020). Systemic cytokines/chemokines associated to radiographic abnormalities in pneumonia in children. Cytokine.

[48] Wang Y., Kong F., Yang Y., Gilbert G.L. (2008). A multiplex PCR-based reverse line blot hybridization (mPCR/RLB) assay for detection of bacterial respiratory pathogens in children with pneumonia. Pediatr Pulmonol.

[49] Zhao Y. 多重PCR技术在婴幼儿肺炎细菌病原学诊断中的临床应用研究: 广东医科大学; 2018.

[50] The Pneumonia Etiology Research for Child Health (PERCH) Study Group (2019). Causes of severe pneumonia requiring hospital admission in children without HIV infection from Africa and Asia: the PERCH multi-country case-control study. Lancet.

[51] Camacho G., Duarte C., García D. (2021). Sentinel surveillance for bacterial pneumonia and meningitis in children under the age of 5 in a tertiary pediatric hospital in Colombia-2016. Biomédica.

[52] Yadav R.K., Kumar D., Singh A., Ziauddin M., Singh D.K. (2021). Clinical and microbial spectrum of community-acquired pneumonia in children of north India. Trop Dr.

[53] García-Elorriaga G., Del Rey-Pineda G. (2016). Basic concepts on community-acquired bacterial pneumonia in pediatrics. Pediatr Infect Dis.

[54] Hammitt L.L., Feikin D.R., Scott J.A.G. (2017). Addressing the analytic challenges of cross-sectional pediatric pneumonia etiology data. Clin Infect Dis.

[55] Feikin D.R., Hammitt L.L., Murdoch D.R., O'Brien K.L., Scott J.A.G (2017). The enduring challenge of determining pneumonia etiology in children: considerations for future research priorities. Clin Infect Dis.

[56] Murdoch D.R., O'Brien K.L., Driscoll A.J. (2012). Laboratory methods for determining pneumonia etiology in children. Clin Infect Dis.

[57] Taylor A.W., Blau D.M., Bassat Q. (2020). Initial findings from a novel population-based child mortality surveillance approach: a descriptive study. Lancet Glob Health.

[58] Driscoll A.J., Deloria Knoll M., Hammitt L.L. (2017). The effect of antibiotic exposure and specimen volume on the detection of bacterial pathogens in children with pneumonia. Clin Infect Dis.

[59] Zar H.J., Andronikou S., Nicol M.P. (2017). Advances in the diagnosis of pneumonia in children. BMJ.

[60] Graham S.M., English M., Hazir T., Enarson P., Duke T. (2008). Challenges to improving case management of childhood pneumonia at health facilities in resource-limited settings. Bull World Health Organ.

[61] Chisti M.J., Tebruegge M., La Vincente S., Graham S.M., Duke T. (2009). Pneumonia in severely malnourished children in developing countries–mortality risk, aetiology and validity of WHO clinical signs: a systematic review. Trop Med Int Health.

[62] McNeil J.C. (2014). Staphylococcus aureus - antimicrobial resistance and the immunocompromised child. Infect Drug Resist.

[63] McNeil J.C. (2014). Staphylococcus aureus–antimicrobial resistance and the immunocompromised child. Infect Drug Resist.

[64] Jukes L., Mikhail J., Bome-Mannathoko N. (2010). Rapid differentiation of staphylococcus aureus, Staphylococcus epidermidis and other coagulase-negative staphylococci and meticillin susceptibility testing directly from growth-positive blood cultures by multiplex real-time PCR. J Med Microbiol.

[65] Asante J., Amoako D.G., Abia A.L. (2020). Review of clinically and epidemiologically relevant coagulase-negative Staphylococci in Africa. Microb Drug Resist.

[66] Fisk A. (1940). The technique of the coagulase test for staphylococci. Br J Exp Pathol.

[67] Ning G., Wang X., Wu D. (2017). The etiology of community-acquired pneumonia among children under 5 years of age in mainland China, 2001–2015: a systematic review. Hum Vaccine Immunother.

[68] Sigaúque B., Roca A., Mandomando I. (2009). Community-acquired bacteremia among children admitted to a rural hospital in Mozambique. Pediatr Infect Dis J.

