# Supplementary material for: The global burden of hospitalisation due to pneumonia caused by Staphylococcus aureus in the under-5 years children: A systematic review and meta-analysis
Source: eClinicalMedicine. 2022 Jan 14;44:101267. doi: 10.1016/j.eclinm.2021.101267 (PMC8763635; doi:10.1016/j.eclinm.2021.101267)
Supplement: Supplementary file 1 [file mmc1.docx]

# Caption for supplementary material

Appendix 1: Search strategies for English database searches

Appendix 2: Search strategies for Chinese database searches

Appendix 3: Eligibility criteria for selection of studies

Appendix 4: List of included studies

Appendix 5: Characteristics of included studies

Figure S5a. Number of studies with non-severe, severe, and very severe pneumonia (n=35)

Figure S5b. Number of studies with community-acquired penumonia and nosocomial pneumonia (n=35)

Appendix 6: Influential plots for meta-analysis of the burden of hospitalisation

Appendix 7: Staphylococcus aureus bacteriology in included studies

Appendix 8: Quality appraisal checklist and assessment of included studies

Appendix 9: Forest plot for subgroup analysis by level of care delivered by health centre
