# Supplementary material for: The global burden of hospitalisation due to pneumonia caused by Staphylococcus aureus in the under-5 years children: A systematic review and meta-analysis
Source: eClinicalMedicine. 2022 Jan 14;44:101267. doi: 10.1016/j.eclinm.2021.101267 (PMC8763635; doi:10.1016/j.eclinm.2021.101267)
Supplement: Supplementary file 2 [file mmc2.docx]

# **Supplementary materials**

Contents

[**Supplementary materials** 1](#_Toc90292187)

[Appendix 1: Search strategies for English database searches 2](#_Toc90292188)

[Appendix 2: Search strategies for Chinese database searches 5](#_Toc90292189)

[Appendix 3: Eligibility criteria for selection of studies 6](#_Toc90292190)

[Appendix 4: List of included studies 8](#_Toc90292191)

[Appendix 5: Characteristics of included studies 10](#_Toc90292192)

[Figure S5a. Number of studies with non-severe, severe, and very severe pneumonia (n=35) 10](#_Toc90292193)

[Figure S5b. Number of studies with community-acquired penumonia and nosocomial pneumonia (n=35) 10](#_Toc90292194)

[Appendix 6: Influential plots for meta-analysis of the burden of hospitalisation 11](#_Toc90292195)

[Appendix 7: *Staphylococcus aureus* bacteriology in included studies 12](#_Toc90292196)

[Appendix 8: Quality appraisal checklist and assessment of included studies 15](#_Toc90292197)

[Appendix 9: Forest plot for subgroup analysis by level of care delivered by health centre 17](#_Toc90292198)

# Appendix 1: Search strategies for English database searches

- MEDLINE (1946 to October Week 4 2020)

1. (aetiology or etiology or aetiological or etiological or organism or pathogen* or causative or causal or agent* or induced or epidemiology).mp. [mp=title, abstract, original title, name of substance word, subject heading word, floating sub-heading word, keyword heading word, organism supplementary concept word, protocol supplementary concept word, rare disease supplementary concept word, unique identifier, synonyms]

2. (pneumonia or lower respiratory infection* or lower respiratory tract infection* or lri or lrti).mp. [mp=title, abstract, original title, name of substance word, subject heading word, floating sub-heading word, keyword heading word, organism supplementary concept word, protocol supplementary concept word, rare disease supplementary concept word, unique identifier, synonyms]

3. (children or child or childhood or pediatric* or paediatric* or infant* or babies or baby or under 5 or under5 or under 5 years).mp. [mp=title, abstract, original title, name of substance word, subject heading word, floating sub-heading word, keyword heading word, organism supplementary concept word, protocol supplementary concept word, rare disease supplementary concept word, unique identifier, synonyms]

4. bacteri*.mp.

5. 1 and 2 and 3 and 4

6. limit 5 to (english language and yr="1990 -Current")

- Global health

1. (aetiology or etiology or aetiological or etiological or organism or pathogen* or causative or causal or agent* or induced or epidemiology).mp. [mp=title, abstract, original title, name of substance word, subject heading word, floating sub-heading word, keyword heading word, organism supplementary concept word, protocol supplementary concept word, rare disease supplementary concept word, unique identifier, synonyms]

2. (pneumonia or lower respiratory infection* or lower respiratory tract infection* or lri or lrti).mp. [mp=title, abstract, original title, name of substance word, subject heading word, floating sub-heading word, keyword heading word, organism supplementary concept word, protocol supplementary concept word, rare disease supplementary concept word, unique identifier, synonyms]

3. (children or child or childhood or pediatric* or paediatric* or infant* or babies or baby or under 5 or under5 or under 5 years).mp. [mp=title, abstract, original title, name of substance word, subject heading word, floating sub-heading word, keyword heading word, organism supplementary concept word, protocol supplementary concept word, rare disease supplementary concept word, unique identifier, synonyms]

4. bacteri*.mp.

5. 1 and 2 and 3 and 4

6. limit 5 to (english language and yr="1990 -Current")

- Embase (1980 to 2020 Week 43)

1. (aetiology or etiology or aetiological or etiological or organism or pathogen* or causative or causal or agent* or induced or epidemiology).mp. [mp=title, abstract, original title, name of substance word, subject heading word, floating sub-heading word, keyword heading word, organism supplementary concept word, protocol supplementary concept word, rare disease supplementary concept word, unique identifier, synonyms]

2. (pneumonia or lower respiratory infection* or lower respiratory tract infection* or lri or lrti).mp. [mp=title, abstract, original title, name of substance word, subject heading word, floating sub-heading word, keyword heading word, organism supplementary concept word, protocol supplementary concept word, rare disease supplementary concept word, unique identifier, synonyms]

3. (children or child or childhood or pediatric* or paediatric* or infant* or babies or baby or under 5 or under5 or under 5 years).mp. [mp=title, abstract, original title, name of substance word, subject heading word, floating sub-heading word, keyword heading word, organism supplementary concept word, protocol supplementary concept word, rare disease supplementary concept word, unique identifier, synonyms]

4. bacteri*.mp.

5. 1 and 2 and 3 and 4

6. limit 5 to (english language and yr="1990 -Current")

- CINAHL

"TX ( aetiology or etiology or aetiological or etiological or organism or causative or causal or agent* or induced or epidemiology ) AND MW ( pneumonia or lower respiratory infection* or lower respiratory tract infection* or lri or lrti ) AND TX ( children or child or childhood or pediatric* or paediatric* or infant* or babies or baby or under 5 or under5 or under 5 years ) AND TX ( incidence or prevalence or case fatality or death* or mortality or hospitalisation* or hospitalization* or hospital stay* or hospital admission* or hospital* ) AND TX bacteri* Publication Year: 1990-2020

- Global Index Medicus

( aetiology OR etiology OR aetiological OR etiological OR organism OR causative OR causal OR agent* OR induced OR epidemiology ) AND ( pneumonia OR lower respiratory tract infection* OR lower respiratory infection* OR lri OR lrti ) AND ( children OR child OR childhood OR pediatric* OR paediatric* OR infant* OR babies OR baby OR under 5 OR under5 OR under 5 years ) AND ( incidence OR prevalence OR case fatality OR death* OR mortality OR hospitalisation* OR hospitalization* OR hospital stay* OR hospital admission* OR hospital* ) AND bacteri*

Filters applied:

English language

Year range: 1990-2020

- Scopus search

( ALL ( aetiology OR etiology OR aetiological OR etiological OR organism OR causative OR causal OR agent* OR induced OR epidemiology ) AND TITLE-ABS-KEY ( pneumonia OR lower AND respiratory AND infection* OR lower AND respiratory AND infection* OR lri OR lrti ) AND ALL ( children OR child OR childhood OR pediatric* OR paediatric* OR infant* OR babies OR baby OR under 5 OR under5 OR under 5 years ) AND ALL ( incidence OR prevalence OR case AND fatality OR death* OR mortality OR hospitalisation* OR hospitalization* OR hospital AND stay* OR hospital AND admission* OR hospital* ) AND ALL ( bacteri* ) ) AND ( LIMIT-TO ( LANGUAGE , "English" ) ) AND ( LIMIT-TO ( PUBYEAR , 2020 ) OR LIMIT-TO ( PUBYEAR , 2019 ) OR LIMIT-TO ( PUBYEAR , 2018 ) OR LIMIT-TO ( PUBYEAR , 2017 ) OR LIMIT-TO ( PUBYEAR , 2016 ) OR LIMIT-TO ( PUBYEAR , 2015 ) OR LIMIT-TO ( PUBYEAR , 2014 ) OR LIMIT-TO ( PUBYEAR , 2013 ) OR LIMIT-TO ( PUBYEAR , 2012 ) OR LIMIT-TO ( PUBYEAR , 2011 ) OR LIMIT-TO ( PUBYEAR , 2010 ) OR LIMIT-TO ( PUBYEAR , 2009 ) OR LIMIT-TO ( PUBYEAR , 2008 ) OR LIMIT-TO ( PUBYEAR , 2007 ) OR LIMIT-TO ( PUBYEAR , 2006 ) OR LIMIT-TO ( PUBYEAR , 2005 ) OR LIMIT-TO ( PUBYEAR , 2004 ) OR LIMIT-TO ( PUBYEAR , 2003 ) OR LIMIT-TO ( PUBYEAR , 2002 ) OR LIMIT-TO ( PUBYEAR , 2001 ) OR LIMIT-TO ( PUBYEAR , 2000 ) OR LIMIT-TO ( PUBYEAR , 1999 ) OR LIMIT-TO ( PUBYEAR , 1998 ) OR LIMIT-TO ( PUBYEAR , 1997 ) OR LIMIT-TO ( PUBYEAR , 1996 ) OR LIMIT-TO ( PUBYEAR , 1995 ) OR LIMIT-TO ( PUBYEAR , 1994 ) OR LIMIT-TO ( PUBYEAR , 1993 ) OR LIMIT-TO ( PUBYEAR , 1992 ) OR LIMIT-TO ( PUBYEAR , 1991 ) OR LIMIT-TO ( PUBYEAR , 1990 ) )

# Appendix 2: Search strategies for Chinese database searches

CNKI

1. Topic = pneumonia or bronchiolitis or respiratory infection

2. Topic = bacteria or pathogen or etiology

3. Topic = child or infant

4. Topic = epidemiology or distribution

1 AND 2 AND 3 AND 4

1990-01-01 to 2020-11-09

Including academic papers and thesis

Wanfang

1. Topic = pneumonia or bronchiolitis or respiratory infection

2. Topic = bacteria or pathogen

3. Topic = child or infant

4. Topic = epidemiology or etiology

1 AND 2 AND 3 AND 4

1990-01-01 to 2020-11-09

Including academic papers and thesis.

CQvip

1. Topic = pneumonia or bronchiolitis or respiratory infection

2. Topic = bacteria or pathogen

3. Topic = child or infant

4. Topic = epidemiology or etiology

1 AND 2 AND 3 AND 4

1990-01-01 to 2020-11-09

# Appendix 3: Eligibility criteria for selection of studies

|  | *Inclusion* | *Exclusion* |
| --- | --- | --- |
| *Participants* | Participants aged ≤ 60 months.  >50% of participants having a sterile site sample tested for bacterial pathogens. | Participants aged > 60 months.  Exclusive neonatal population studies.  ≤50% samples having a sterile site sample tested for bacterial pathogens.  Complicated pneumonia or recurrent pneumonia.  Chronic respiratory conditions like asthma, cystic fibrosis, etc.  Neurological, cardiovascular, and other respiratory conditions. |
| *Health condition* | Pneumonia,  acute lower respiratory tract infection (ALRI),  lower respiratory tract infection (LRI). | Acute respiratory infection (ARI),  middle ear infections |
| *Outcome** | Number of samples with *Staphylococcus aureus* from a sterile site. | Sample not tested for *Staphylococcus aureus*.  No data on number of samples with *Staphylococcus aureus*. |
| *Comparator* | Number of samples with other bacteria detected and with no bacterial pathogens detected. | Number of samples with other bacteria or no bacteria not reported. |
| *Study design* | Observational studies- longitudinal cross-sectional surveys, cohort studies, case-control studies, prevalence surveys, point-prevalence surveys, diagnostic testing efficacy studies.  Intervention studies if bacterial pathogens tested before intervention administration. | Intervention studies if bacterial pathogens were tested after administration of intervention.  Editorials  Letters to editor  Secondary literature: non-systematic literature reviews and systematic reviews. |
| *Case definitions* | WHO case definition of pneumonia or acute lower respiratory infections (ALRI), WHO radiographic criteria of pneumonia, pneumonia diagnosed based on clinical signs and symptoms or radiological evidence by a medical professional.  Both community-acquired pneumonia and hospital-acquired pneumonia cases**. | Respiratory illness other than pneumonia |
| *Samples* | Sterile-site samples: blood, induced sputum, bronchoalveolar lavage, pleural fluid, bronchial aspirate, lung fluid, cerebrospinal fluid | Nasopharyngeal samples |
| *Tests* | Culture, polymerase chain reaction (PCR) |  |
| *Settings* | In-patient or hospital setting.  All countries. | Out-patient setting. |
| *Timeframe* | Data collection in or after year 1990. | Data collection before 1990. |
| *Language* | Studies published in English language  Studies published in Chinese language. | Studies published in languages other than English or Chinese. |
| *Studies were selected if they reported the primary outcome and irrespective of the secondary outcome  **The selection criteria did not exclude nosocomial pneumonia cases. However, none of the studies reporting data explicitly on nosocomial pneumonia fulfilled all the other criteria. | | |

# Appendix 4: List of included studies

| First Author name | Published year | Study title |
| --- | --- | --- |
| Abdelkhalig, S.M. | 2015 | Viral and bacterial acute lower respiratory tract infections in Khartoum children emergency hospital in 2012 |
| Adegbola, R.A. | 1994 | The etiology of pneumonia in malnourished and well-nourished Gambian children |
| Asghar, R. | 2008 | Chloramphenicol versus ampicillin plus gentamicin for community acquired very severe pneumonia among children aged 2-59 months in low resource settings: multicentre randomised controlled trial (SPEAR study) |
| Bahl, R. | 1995 | A bacteriological study in hospitalized children with pneumonia |
| Baqui, A. H. | 2007 | A Population-based Study of Hospital Admission Incidence Rate and Bacterial Aetiology of Acute Lower Respiratory Infections in Children Aged Less Than Five Years in Bangladesh |
| Bari, A. | 2014 | Disease Pattern And Bacteriological Profile Of Childhood Pneumonia |
| Barrett, C. | 2016 | Differences Between Radiologically Confirmed Pneumonia With and Without Pleural Fluid in Hospitalized Children Younger Than 5 Years in Southern Israel |
| Bautista-Marquez, A. | 2013 | Prevalence of pneumococcal disease, serotype distribution, and antimicrobial susceptibility in Mexican children younger than 5 years of age |
| Benet, T. | 2017 | Severity of pneumonia in under 5-year-old children from developing countries: a multicenter, prospective, observational study |
| Benet, T. | 2017 | Microorganisms associated with pneumonia in children <5 years of age in developing and emerging countries: the GABRIEL pneumonia multicenter, prospective, case-control study |
| Camacho-Moreno, G. | 2021 | Sentinel surveillance for bacterial pneumonia and meningitis in children under the age of 5 in a tertiary pediatric hospital in Colombia - 2016 |
| Capeding, M.R. | 1994 | Etiology of acute lower respiratory infection in Filipino children under five years |
| Champatiray, J. | 2017 | Clinico-Aetiological Study of Severe and Very Severe Pneumonia in Two Months to Five Years Children in a Tertiary Health Care Centre in Odisha, India |
| Ekalaksananan, T. | 2001 | Etiology of acute lower respiraotry tract infection in children at Srinagarind hospital, Khon Kaen, Thailand |
| El Mdaghri, N. | 2012 | Epidemiological profile of invasive bacterial diseases in children in Casablanca, Morocco: antimicrobial susceptibilities and serotype distribution |
| Hammitt, L. | 2012 | A preliminary study of pneumonia etiology among hospitalized children in Kenya |
| Hasan, K. | 2006 | Viral etiology of pneumonia in a cohort of newborns till 24 months of age in Rural Mirzapur, Bangladesh |
| Hijazi, Z. | 1997 | Acute lower respiratory tract infections in children in Kuwait |
| Howie, S.R.C. | 2014 | Etiology of Severe Childhood Pneumonia in The Gambia, West Africa, Determined by Conventional and Molecular Microbiological Analyses of Lung and Pleural Aspirate Samples |
| Jakhar S. K. | 2018 | Etiology and risk factors determining poor outcome of severe pneumonia in under-five children |
| Kurade, A. | 2018 | Induced Sputum as a Diagnostic Tool in Pneumonia in Under Five Children—A Hospital-based Study |
| Madhi, S.A. | 2000 | Increased Disease Burden and Antibiotic Resistance of Bacteria Causing Severe Community-Acquired Lower Respiratory Tract Infections in Human Immunodeficiency Virus Type 1–Infected Children |
| Moreno, L. | 2006 | Development and Validation of a Clinical Prediction Rule to Distinguish Bacterial From Viral Pneumonia in Children |
| Nantanda, R. | 2008 | Bacterial aetiology and outcome in children with severe pneumonia in Uganda |
| Nathan, A. M. | 2020 | Bacterial pneumonia and its associated factors in children from a developing country: A prospective cohort study |
| Ngocho, J. S. | 2020 | Inappropriate treatment of community-acquired pneumonia among children under five years of age in Tanzania |
| Onipede, A.O. | 2009 | Invasive bacteria isolates from children with severe infections in a Nigerian hospital |
| PERCH | 2019 | Basse, Bamako, Lusaka, Soweto, Kilifi, Dhaka and Matlab, Nakhon Phanom and Sa Kaeo |
| Schwarz, N.G. | 2010 | Systemic bacteraemia in children presenting with clinical pneumonia and the impact of non-typhoid salmonella (NTS) |
| Sigau´que, B. | 2009 | Severe pneumonia in Mozambican young children: clinical and radiological characteristics and risk factors |
| Thea, D.M. | 2017 | Limited utility of polymerase chain reaction in induced sputum specimens for determining the causes of childhood pneumonia in resource-poor settings: Findings from the pneumonia etiology research for child health (PERCH) study |
| Vasconcellos, A.G. | 2020 | Systemic cytokines/chemokines associated to radiographic abnormalities in pneumonia in children |
| Wang, Y. | 2008 | A multiplex PCR-based reverse line blot hybridization (mPCR/RLB) assay for detection of bacterial respiratory pathogens in children with pneumonia |
| Yadav, R. | 2021 | Clinical and microbial spectrum of community-acquired pneumonia in children of north India |
| Zhao | 2018 | 多重PCR技术在婴幼儿肺炎细菌病原学诊断中的临床应用研究 |

# Appendix 5: Characteristics of included studies

### Figure S5a. Number of studies with non-severe, severe, and very severe pneumonia (n=35)

### Figure S5b. Number of studies with community-acquired penumonia and nosocomial pneumonia (n=35)

# Appendix 6: Influential plots for meta-analysis of the burden of hospitalisation

| 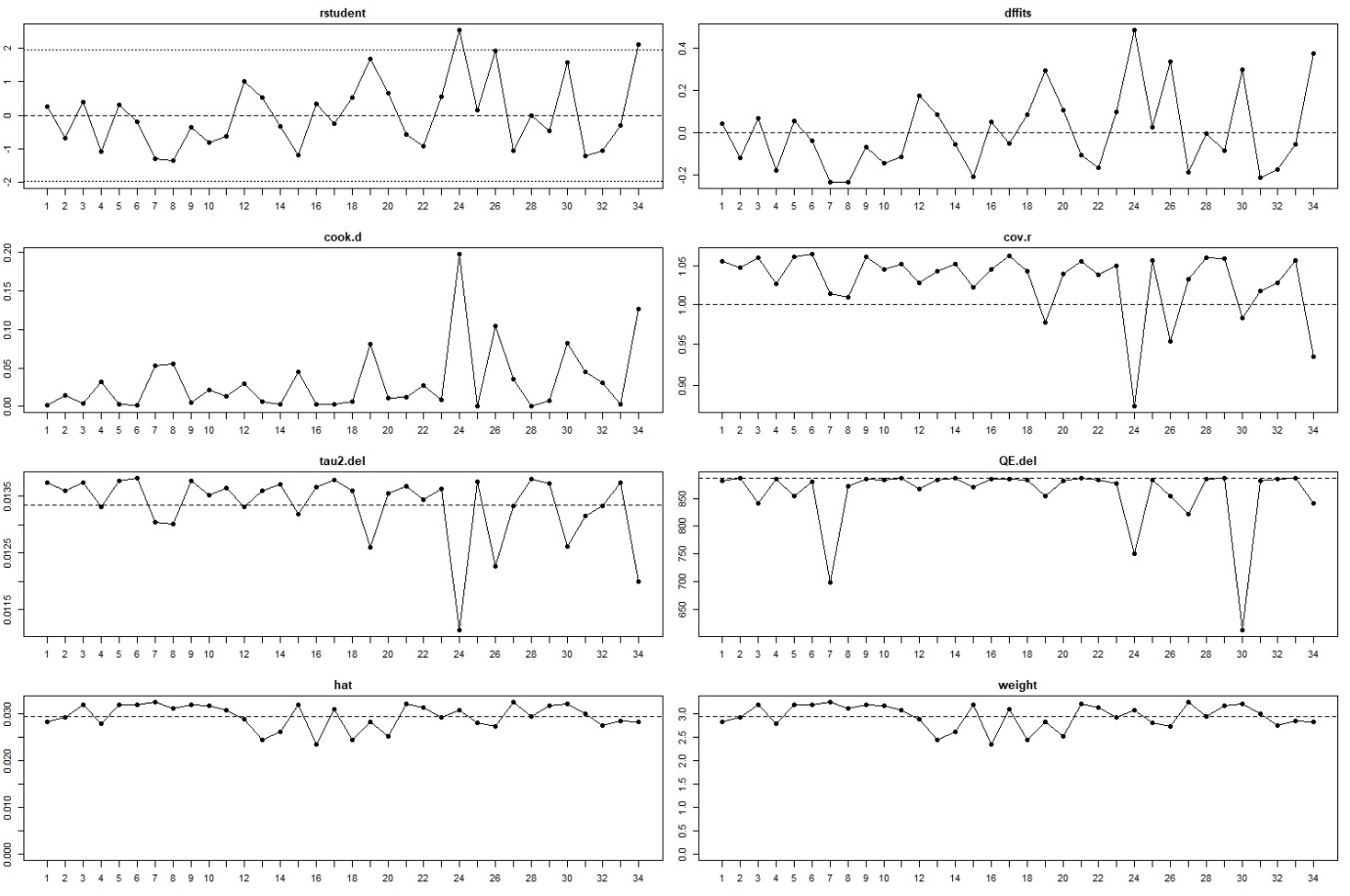 |
| --- |
| rstudent= studentised deleted residual  dffits= DIFFITS value  cook.d= Cook’s distance  cov.r= Covariance ratio  tau2.del= τ 2 after omission of the study  QE.del= χ 2 test statistic for homogeneity after omission of the study  hat= hat values indicating influence of study on the meta-analytic effect size  weight= weight of study in the overall meta-analytic average effect siz |

# Appendix 7: *Staphylococcus aureus* bacteriology in included studies

**Classification of Culture Methods**

Use of Preferred Media [SCORE 2]

- Tryptic soy agar [TSA]
- Brain Heart Infusion broth [BHI]
- Luria Bertani Agar [LB]

Details of other media used but not the above preferred media; this includes automated blood culture systems [SCORE 1]

No details of media used [SCORE 0]

**Classification of methods of identification**

Use of “standard methods” plus coagulase test OR sequencing OR PCR [SCORE 2]

Use of standard methods with further tests such as for those below [SCORE 1]

- Catalase activity
- Mannitol fermentation
- Gelatin hydrolysis
- Beta haemolysis

Use of “standard methods” with no further details [SCORE 0]

- For example, Gram stain and morphology [Colonies - clustering with yellow pigment]

Quality assessment of bacteriological methods of included studies

| Author | Classification of culture methods score | Classification of methods of identification score | Total score |
| --- | --- | --- | --- |
| Abdelkhalig (2015) | 1 | 2 | 1 |
| Adegbola (1994) | 2 | 0 | 2 |
| Asghar (2008) | 0 | 0 | 3 |
| Bahl (1995) | 0 | 0 | 3 |
| Baqui (2007) | 2 | 0 | 2 |
| Bari (2014) | 0 | 0 | 3 |
| Barrett (2016) | 0 | 0 | 3 |
| Bautista-Marquez (2013) | 1 | 0 | 3 |
| Benet (a)  (2017) | 0 | 2 | 2 |
| Benet (b) (2017) | 0 | 2 | 2 |
| Camacho-Moreno, (2021) | 1 | 0 | 3 |
| Capeding (1994) | 0 | 0 | 3 |
| Champatiray (2017) | 1 | 0 | 3 |
| Ekalaksananan (2001) | 0 | 0 | 3 |
| El mdaghri (2012) | 1 | 0 | 3 |
| Hammitt (2012) | 1 | 0 | 3 |
| Hasan (2006) | 1 | 0 | 3 |
| Hijazi (1997) | 0 | 0 | 3 |
| Howie (2014) | 2 | 2 | 1 |
| Jakhar (2018) | 0 | 0 | 3 |
| Kurade (2018) | 1 | 0 | 3 |
| Madhi (2000) | 1 | 0 | 3 |
| Moreno (2006) | 1 | 0 | 3 |
| Nantanda (2008) | 1 | 0 | 3 |
| Nathan (2020) | 0 | 2 | 2 |
| Ngocho (2020) | 1 | 2 | 1 |
| Onipede (2009) | 2 | 2 | 1 |
| Perch (2019) | 1 | 2 | 1 |
| Schwarz (2010) | 1 | 0 | 3 |
| Sigau´que (2009) | 1 | 0 | 3 |
| Thea (2017) | 1 | 2 | 1 |
| Vasconcellos (2020) | 1 | 0 | 3 |
| Wang (2008) | 0 | 0 | 3 |
| Yadav (2021) | 2 | 0 | 2 |
| Zhao (2018) | 0 | 2 | 2 |

We classified studies into three groups based on quality of bacteriological methods:

1. *Highest quality group* - those with a score of at least 1 for culture methods AND a score of at least one for identification methods interpretation [n=6]
2. *Moderate quality group* - those with a total score [adding the two scores together] of 2 or more but which are not in the highest quality group [n=6]; these all scored 2 and 0

(*n=6 and not 7 because there are two Benet studies. We extracted data for hospitalisation from one study (that had a greater sample size but did not have data on CFR). But we extracted data for CFR from the other study. Therefore, the second Benet study is not included in the hospitalisation analysis).

1. *Lower quality group* - all the other studies [with total scores of o or 1] [n=22]

| ***Forest plot for pooled proportion of Staphylococcus aureus pneumonia in under-5 children hospitalised for pneumonia by subgroup: quality of bacteriological methods: high, medium, and low*** |
| --- |
| 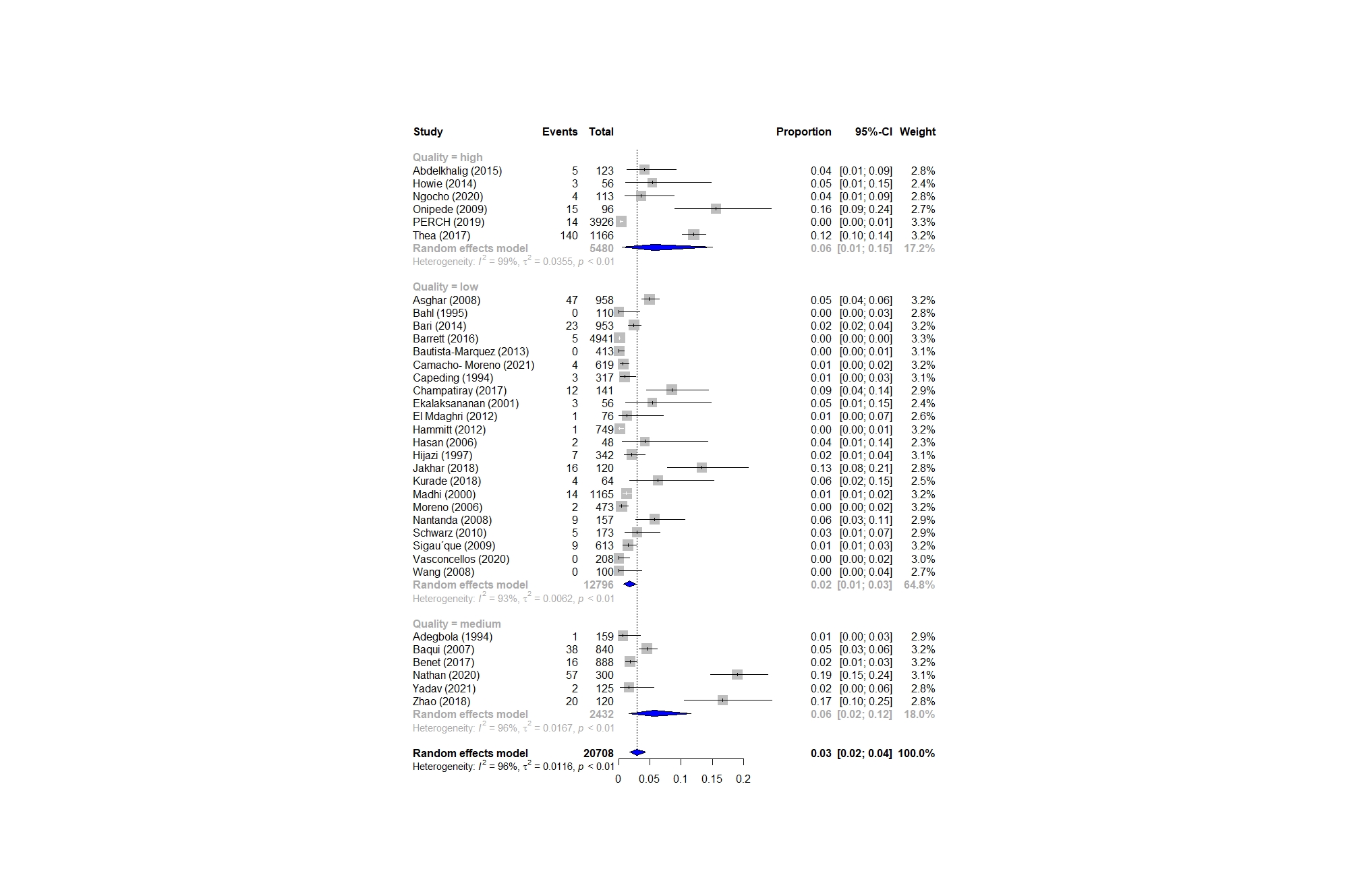 |

# Appendix 8: Quality appraisal checklist and assessment of included studies

| Study number | First author (year) | Was a consecutive or random sample of patients screened for eligibility? | Were at least 80% potential participants identified while screening included in the final analysis? | Was pneumonia clearly defined for the study population? | Were pneumonia cases clearly classified as community-acquired or hospital-acquired? | Were samples collected by trained staff? | Did a majority of participants have at least 1.5ml sample collected for testing? | Were samples collected before the administration of antibiotics? | Were appropriate measures undertaken to avoid cross-contamination of samples during collection? | Were appropriate measures undertaken to avoid cross-contamination of samples during transport/ storage? | Was a coagulase test or PCR conducted? | Were data collected on the nutrition status of the participants? | Were data collected on the HIV status of participants? | Total score |
| --- | --- | --- | --- | --- | --- | --- | --- | --- | --- | --- | --- | --- | --- | --- |
| 1 | Abdelkhalig (2015) | no | yes | yes | no | unclear | unclear | unclear | yes | unclear | yes | yes | no | 5 |
| 2 | Adegbola (1994) | unclear | yes | no | yes | unclear | unclear | no | yes | unclear | no | yes | no | 4 |
| 3 | Asghar (2008) | yes | yes | yes | yes | yes | unclear | no | unclear | unclear | no | yes | yes | 7 |
| 4 | Bahl (1995) | yes | yes | yes | no | unclear | unclear | no | unclear | unclear | no | yes | no | 4 |
| 5 | Baqui (2007) | yes | no | yes | yes | unclear | unclear | no | unclear | unclear | no | no | no | 3 |
| 6 | Bari (2014) | yes | yes | yes | no | unclear | unclear | no | unclear | unclear | no | no | no | 3 |
| 7 | Barrett (2016) | yes | yes | yes | no | unclear | unclear | no | unclear | unclear | no | no | no | 3 |
| 8 | Bautista-Marquez (2013) | yes | yes | yes | yes | unclear | unclear | unclear | unclear | unclear | no | no | no | 4 |
| 9 | Benet  (2017a) | yes | yes | yes | yes | unclear | unclear | no | unclear | unclear | yes | yes | yes | 7 |
| 10 | Benet (2017b) | yes | yes | yes | yes | unclear | unclear | no | unclear | unclear | yes | yes | yes | 7 |
| 11 | Camacho-Moreno (2021) | yes | yes | yes | no | unclear | unclear | unclear | unclear | unclear | no | no | no | 3 |
| 12 | Capeding (1994) | yes | yes | no | no | unclear | unclear | no | unclear | unclear | no | yes | no | 3 |
| 13 | Champatiray (2017) | yes | yes | yes | no | unclear | yes | unclear | yes | unclear | no | yes | no | 6 |
| 14 | Ekalaksananan (2001) | yes | yes | yes | yes | unclear | unclear | unclear | unclear | unclear | no | no | no | 4 |
| 15 | El mdaghri (2012) | yes | yes | yes | no | unclear | unclear | unclear | unclear | unclear | no | no | no | 3 |
| 16 | Hammitt (2012) | no | no | yes | no | unclear | unclear | no | unclear | unclear | no | yes | yes | 3 |
| 17 | Hasan (2006) | yes | no | yes | yes | unclear | yes | yes | yes | unclear | no | no | no | 6 |
| 18 | Hijazi (1997) | unclear | yes | yes | yes | unclear | unclear | no | unclear | unclear | no | yes | no | 4 |
| 19 | Howie (2014) | unclear | yes | yes | no | unclear | unclear | unclear | unclear | unclear | yes | no | yes | 4 |
| 20 | Jakhar (2018) | yes | yes | yes | yes | unclear | unclear | unclear | unclear | unclear | no | yes | no | 5 |
| 21 | Kurade (2018) | unclear | no | yes | no | yes | unclear | no | unclear | unclear | no | no | no | 2 |
| 22 | Madhi (2000) | yes | yes | yes | yes | unclear | unclear | no | unclear | unclear | no | yes | yes | 6 |
| 23 | Moreno (2006) | yes | yes | no | yes | unclear | unclear | no | unclear | unclear | no | no | no | 3 |
| 24 | Nantanda (2008) | unclear | yes | yes | no | unclear | yes | no | unclear | unclear | no | yes | yes | 5 |
| 25 | Nathan (2020) | yes | no | yes | yes | unclear | unclear | no | unclear | yes | yes | yes | no | 6 |
| 26 | Ngocho (2020) | yes | yes | yes | yes | yes | unclear | no | yes | yes | no | yes | yes | 9 |
| 27 | Onipede (2009) | yes | yes | yes | no | unclear | unclear | no | yes | unclear | yes | no | no | 5 |
| 28 | Perch (2019) | no | yes | yes | yes | unclear | yes | no | unclear | unclear | no | yes | yes | 6 |
| 29 | Schwarz (2010) | unclear | unclear | yes | no | unclear | unclear | no | unclear | unclear | no | no | yes | 2 |
| 30 | Sigau´que (2009) | yes | yes | yes | yes | unclear | unclear | unclear | unclear | unclear | no | yes | yes | 6 |
| 31 | Thea (2017) | unclear | no | yes | yes | yes | unclear | no | unclear | yes | yes | yes | yes | 7 |
| 32 | Vasconcellos (2020) | yes | yes | no | yes | unclear | unclear | unclear | unclear | unclear | no | no | yes | 4 |
| 33 | Wang (2008) | unclear | yes | yes | yes | unclear | unclear | unclear | unclear | unclear | no | no | no | 3 |
| 34 | Yadav (2021) | yes | yes | yes | yes | unclear | yes | yes | unclear | unclear | no | no | no | 6 |
| 35 | Zhao (2018) | unclear | no | yes | no | unclear | unclear | unclear | unclear | yes | yes | no | no | 3 |

# Appendix 9: Forest plot for subgroup analysis by level of care delivered by health centre

| 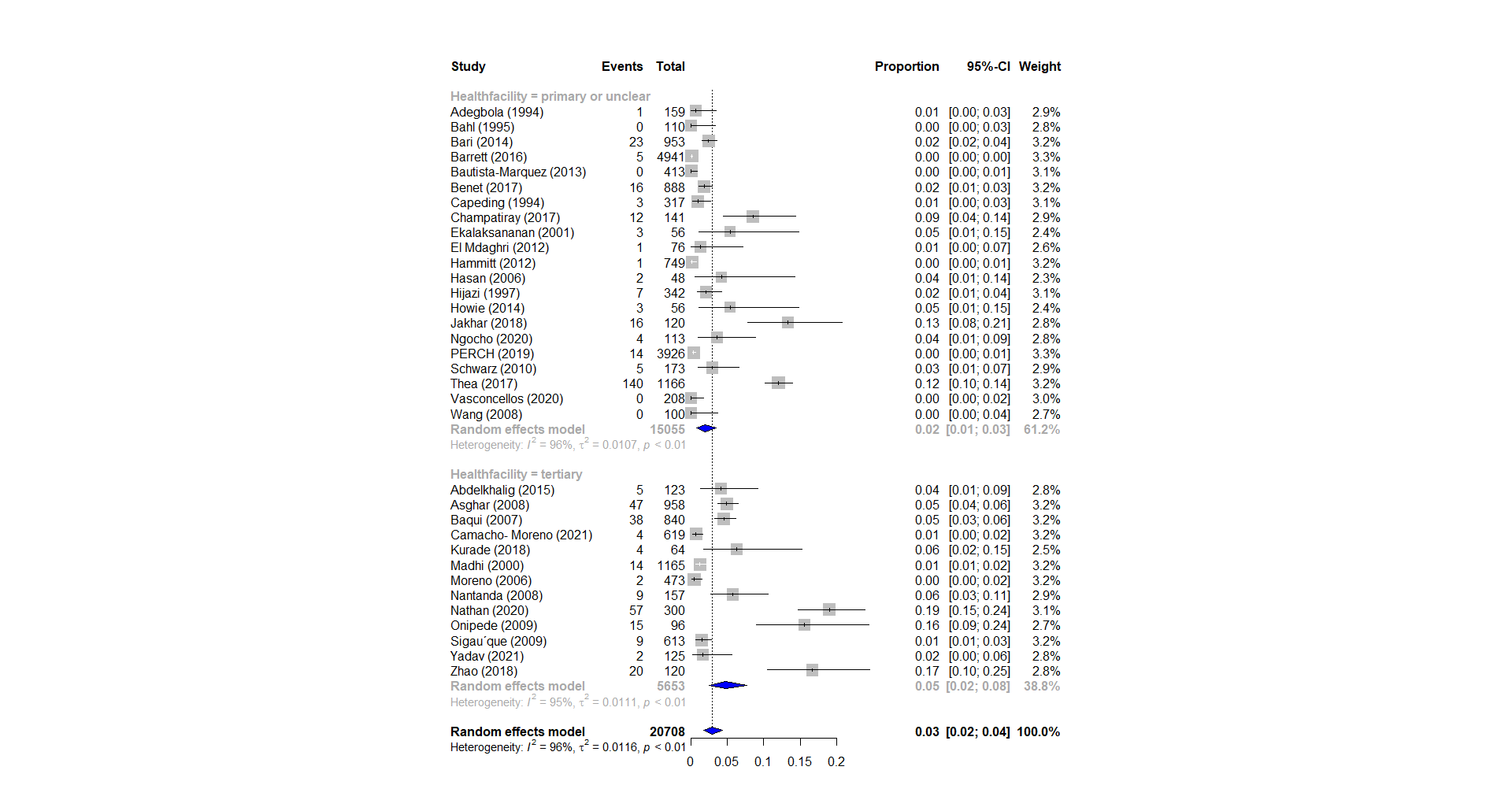 |
| --- |
